# Smoking, use of smokeless tobacco, HLA genotypes and incidence of latent autoimmune diabetes in adults

**DOI:** 10.1007/s00125-022-05763-w

**Published:** 2022-07-28

**Authors:** Jessica Edstorp, Yuxia Wei, Emma Ahlqvist, Lars Alfredsson, Valdemar Grill, Leif Groop, Bahareh Rasouli, Elin P. Sørgjerd, Per M. Thorsby, Tiinamaija Tuomi, Bjørn O. Åsvold, Sofia Carlsson

**Affiliations:** 1 Institute of Environmental Medicine, Karolinska Institutet, Stockholm, Sweden; 2 Department of Clinical Sciences in Malmö, Clinical Research Centre, Lund University, Malmö, Sweden; 3 Center for Occupational and Environmental Medicine, Region Stockholm, Stockholm, Sweden; 4 Department of Clinical and Molecular Medicine, Norwegian University of Science and Technology, Trondheim, Norway; 5 Institute for Molecular Medicine Finland, Helsinki University, Helsinki, Finland; 6 Department of Global Health and Population, Harvard TH Chan School of Public Health, Boston, MA USA; 7 HUNT Research Centre, Department of Public Health and Nursing, NTNU, Norwegian University of Science and Technology, Trondheim, Norway; 8 Department of Endocrinology, Clinic of Medicine, St Olavs Hospital, Trondheim, Norway; 9 Hormone Laboratory, Department of Medical Biochemistry, Oslo University Hospital, Aker, Oslo, Norway; 10 Biochemical Endocrinology and Metabolism Research Group, Oslo University Hospital, Aker, Oslo, Norway; 11 Division of Endocrinology, Abdominal Center, Helsinki University Hospital, Helsinki, Finland; 12 Research Program for Diabetes and Obesity, University of Helsinki, Helsinki, Finland; 13 Folkhälsan Research Center, Helsinki, Finland; 14 K.G. Jebsen Center for Genetic Epidemiology, Department of Public Health and Nursing, NTNU, Norwegian University of Science and Technology, Trondheim, Norway

**Keywords:** Gene–environment interaction, LADA, Latent autoimmune diabetes in adults, Mendelian randomisation analysis, Smoking, Tobacco use

## Abstract

**Aims/hypotheses:**

Smoking and use of smokeless tobacco (snus) are associated with an increased risk of type 2 diabetes. We investigated whether smoking and snus use increase the risk of latent autoimmune diabetes in adults (LADA) and elucidated potential interaction with HLA high-risk genotypes.

**Methods:**

Analyses were based on Swedish case–control data (collected 2010–2019) with incident cases of LADA (*n*=593) and type 2 diabetes (*n*=2038), and 3036 controls, and Norwegian prospective data (collected 1984–2019) with incident cases of LADA (*n*=245) and type 2 diabetes (*n*=3726) during 1,696,503 person-years of follow-up. Pooled RRs with 95% CIs were estimated for smoking, and ORs for snus use (case–control data only). The interaction was assessed by attributable proportion (AP) due to interaction. A two-sample Mendelian randomisation (MR) study on smoking and LADA/type 2 diabetes was conducted based on summary statistics from genome-wide association studies.

**Results:**

Smoking (RR_pooled_ 1.30 [95% CI 1.06, 1.59] for current vs never) and snus use (OR 1.97 [95% CI 1.20, 3.24] for ≥15 box-years vs never use) were associated with an increased risk of LADA. Corresponding estimates for type 2 diabetes were 1.38 (95% CI 1.28, 1.49) and 1.92 (95% CI 1.27, 2.90), respectively. There was interaction between smoking and HLA high-risk genotypes (AP 0.27 [95% CI 0.01, 0.53]) in relation to LADA. The positive association between smoking and LADA/type 2 diabetes was confirmed by the MR study.

**Conclusions/interpretation:**

Our findings suggest that tobacco use increases the risk of LADA and that smoking acts synergistically with genetic susceptibility in the promotion of LADA.

**Data availability:**

Analysis codes are shared through GitHub (https://github.com/jeseds/Smoking-use-of-smokeless-tobacco-HLA-genotypes-and-incidence-of-LADA).

**Graphical abstract:**

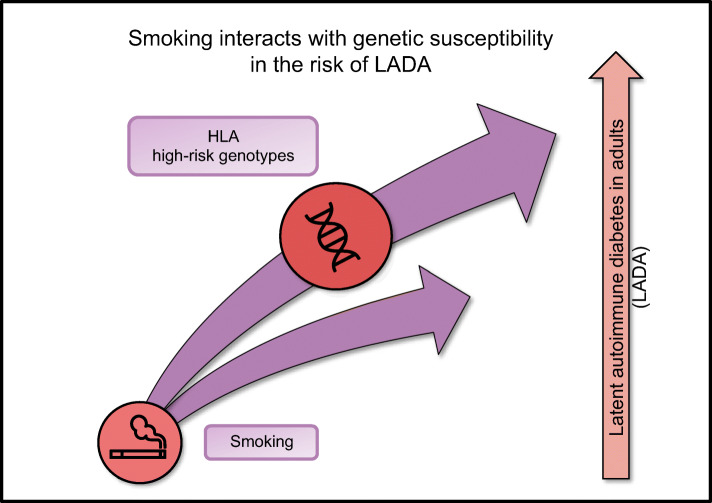

**Supplementary Information:**

The online version contains peer-reviewed but unedited supplementary material available at 10.1007/s00125-022-05763-w.

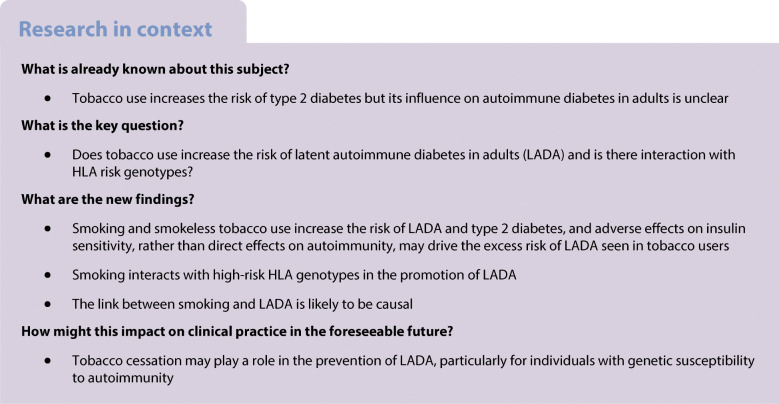



## Introduction

Smoking is associated with an increased risk of type 2 diabetes [1] and a Mendelian randomisation (MR) study supports a causal relationship [2]. The association is primarily attributed to negative effects on insulin sensitivity [3]. In contrast, prenatal exposure to smoking is linked to a reduced risk of type 1 diabetes [4], possibly due to an immunosuppressive effect of nicotine [5].

Latent autoimmune diabetes in adults (LADA) resembles both type 1 and type 2 diabetes. It is characterised by an autoimmune reaction that is milder than in type 1 diabetes and progression towards insulin dependence is slower. Genetic susceptibility is mainly manifested through the type 1 diabetes-associated HLA gene complex [6]. In addition, individuals with LADA share phenotypic characteristics with individuals with type 2 diabetes, including adult onset and insulin resistance [7].

Studies on smoking and the risk of LADA are few and conflicting. We observed a reduced risk in the Trøndelag Health Study (HUNT) in Norway [8] and an increased risk using Swedish case–control data [9]. Since the pathogenesis of LADA includes both autoimmune insulitis and insulin resistance, associations with smoking such as those seen in both type 1 and type 2 diabetes seem biologically plausible. The net effect may depend on genetic susceptibility to LADA so that certain effects of smoking are augmented in those carrying high-risk genotypes. Notably, interaction between HLA genotypes and smoking has been observed in the aetiology of autoimmune rheumatoid arthritis and multiple sclerosis [10, 11], although potential interaction remains to be explored in LADA.

Studies of other types of tobacco in relation to autoimmune diabetes are scarce. Commonly used in Scandinavia, Swedish smokeless tobacco (snus) is high in nicotine but contains fewer of the other harmful components found in cigarettes [12]. Snus use has been linked to an increased risk of type 2 diabetes [13]. The only study in LADA found no association but was hampered by small numbers [14].

Our aim was to assess the hypothesis that tobacco use increases the risk of LADA and to investigate, for the first time, whether tobacco use interacts with HLA high-risk genotypes in the promotion of LADA. For these purposes, we expand on our previous observational studies by using updated data, including newly recruited cases from two Scandinavian population-based studies with incident cases of LADA. We also performed a two-sample MR study to investigate whether the link between smoking and LADA was causal.

## Methods

### The ESTRID study

#### Study population

Epidemiological Study of Risk Factors for LADA and Type 2 Diabetes (ESTRID) is a Swedish population-based case–control study nested within the All New Diabetics in Scania (ANDIS) registry and biobank [15], aimed at characterising all incident diabetes cases in Scania county with regard to clinical and genetic features. Since 2010, ESTRID has enrolled incident cases of LADA and type 2 diabetes from ANDIS. Lifestyle and demographic information is collected by questionnaire, administered close to diagnosis (median 4.9 months). Matched controls are randomly selected from the general population of Scania through incidence-density sampling. These controls respond to the questionnaire but do not provide blood samples for genetic analyses. Therefore, the present study includes population-based, diabetes-free controls from the Epidemiological Investigation of Rheumatoid Arthritis (EIRA) study [16], a case–control study with a similar set-up to that of ESTRID. Rheumatoid arthritis is more common among women than men, as reflected by a larger proportion of women in the control group of this study. For this reason, the ‘genetic’ controls are matched to cases by sex and age.

The present study is based on all cases collected in ESTRID 2010–2019 with information on tobacco use (LADA *n*=593), type 2 diabetes (*n*=2038) and all controls aged ≥35 years included in EIRA 2006–2014 (*n*=3036). All participants gave informed consent, and the study was approved by the ethical review board in Stockholm.

#### Diabetes classification and laboratory analyses

Diabetes cases were diagnosed within the healthcare system of Scania. Fasting glucose and C-peptide were analysed in blood samples drawn at diagnosis [15]. An ELISA (RSR, Cardiff, UK) measured GAD antibodies (GADA) in those diagnosed with diabetes. Sensitivity and specificity were 0.84 and 0.98 [17], respectively, and values above 250 U/ml were censored. Individuals with LADA were aged ≥35 years at diagnosis, GADA positive (≥10 U/ml) and had C-peptide levels ≥0.2 nmol/l (IMMULITE 2000; Siemens Healthcare Diagnostics Product, Llanberis, UK) or ≥0.3 nmol/l (Cobas e601; Roche Diagnostics, Mannheim, Germany). The C-peptide criterion separated LADA from type 1 diabetes. Type 2 diabetes was defined as age ≥35 years, GADA negativity and C-peptide >0.60 nmol/l (IMMULITE) or >0.72 nmol/l (Cobas). HOMA-IR and HOMA-B were based on the relationship between fasting plasma glucose and serum C-peptide [18].

#### Genetic information

DNA samples from the ANDIS biobank were analysed using iPLEX (Sequenom, San Diego, CA, USA) or TaqMan assays (Thermo Fisher Scientific, Carlsbad, CA, USA) [15]. Samples from the EIRA biobank were analysed with the Illumina Global Screening array or an Infinium Illumina 300K immunochip custom array (Illumina, San Diego, CA, USA). Imputation was performed using Minimac4 (https://genome.sph.umich.edu/wiki/Minimac4) based on a Genome Reference Consortium assembly (GRCh37/hg19).

Three SNPs tagging HLA genotypes (rs3104413, rs2854275, rs9273363) predicted *HLA-DRB1* (*DR3/DR4*) and *HLA-DQB1* (*DQ2/DQ8*) with an overall accuracy of 99.3% [19]. High genetic risk was defined as HLA genotypes *DR3/3*, *DR3/4*, *DR4/4*, or haplotypes of *DR4-DQ8* or *DR3-DQ2*; *DR3/X*, *DR4/X*, *DRX/X* (where *X* is neither 3 nor 4) and *DR4-DQ7* were classified as low/intermediate risk genotypes.

### The HUNT study

#### Study population

In Trøndelag County, Norway, the entire population aged ≥20 years have been invited to participate in the HUNT study [20] on four occasions between 1984 and 2019 (HUNT1 1984–1986, HUNT2 1995–1997, HUNT3 2006–2008, HUNT4 2017–2019). The study includes questionnaires, clinical examination and blood sampling. Eligible for analyses were all participants in HUNT1–3 with at least one follow-up (*n*=94,489). After exclusion of those with diabetes at baseline (*n*=2481) or without smoking information (*n*=13,466), the analytical sample consisted of 78,542 individuals. The study was approved by the Norwegian Data Protection Authority and the Regional Committee for Medical and Health Research Ethics and all participants provided informed consent.

#### Diabetes classification and laboratory analyses

Incident diabetes was identified through self-report, which according to a previous study has high validity (95% of self-reports are confirmed by medical records) [21]. Classification was based on age at diagnosis and GADA assessment at follow-up (median 5 years after diagnosis). GADA was analysed at Hormone laboratory, Oslo University Hospital, by immunoprecipitation radioligand assay (Novo Nordisk, Bagsværd, Denmark) in samples collected during HUNT2 and HUNT3. The sensitivity and specificity of the assay were 0.64 and 1.00, respectively (Islet Autoantibody Standardization Program 2003). In HUNT4, GADA was measured using ELISA (RSR) with a sensitivity and specificity of 0.84 and 0.98, respectively (Islet Autoantibody Standardization Program 2020 report), measuring range 5–2000 U/ml, CV 9% at 9.6 U/ml and limit of quantification 5 U/ml. The GADA methods are accredited according to ISO 17025.

All individuals aged ≥35 years with GADA positivity (≥0.08 U/ml in HUNT2 and HUNT3; ≥10 U/ml in HUNT4 [to harmonise with ESTRID cases]) were classified as having LADA (*n*=245) and as having type 2 diabetes if they were GADA negative (*n*=3726). We could not separate LADA from adult-onset type 1 diabetes since C-peptide was not measured at diagnosis and information on treatment was not available for everyone.

#### Genetic information

The blood samples were genotyped for *HLA-DRB1*- and *HLA-DQB1*-associated SNPs at the Norwegian University of Science and Technology Genomic Core Facility, Trondheim, by HumanCoreExome, Illumina (San Diego, CA, USA). Imputation was performed using Minimac3 (v2.0.1, https://genome.sph.umich.edu/wiki/Minimac3) and a customised Haplotype Reference Consortium release 1.1 (HRC v1.1). Two SNPs were available to infer high-risk *DR3-DQ2* (rs2854275) and *DR4-DQ8* haplotypes (rs9273363). All other were classified as low/intermediate risk genotypes.

### Tobacco use and covariates

The ESTRID/EIRA and HUNT questionnaires contained detailed questions regarding lifetime exposure to smoking, including intensity and duration. The ESTRID/EIRA questionnaires contained corresponding questions on snus use. In HUNT, baseline information on snus was only available in HUNT3 and the number of exposures was too low for viable analyses (*n*=5).

Smoking/snus use intensity in current users was categorised as light/moderate (<20 cigarettes per day or <7 boxes of snus per week) or heavy (≥20 cigarettes per day or ≥7 boxes of snus per week). Smoking intensity was also assessed continuously in current smokers. Cumulative use was assessed in ever smokers/snus users, where one pack/box-year equals smoking one pack of cigarettes per day or using seven boxes of snus per week for a year. Index date was set to 1 year prior to diagnosis/participation for ESTRID cases and controls.

In ESTRID, information on alcohol consumption was based on beverage-specific questions regarding the amount and frequency consumed during the preceding year, whereas in HUNT frequency of alcohol consumption related to the past weeks or months. Consumption was assessed categorically, from abstainers to high consumers. Educational level was categorised into low (primary school), medium (upper secondary school) or high (university). BMI (weight [kg]/height [m^2^]) was based on anthropometric measurements in HUNT and self-reported weight and height in ESTRID. The questionnaires also contained information on physical activity and family history of diabetes.

### Two-sample MR study

A typical MR analysis uses uncorrelated genetic variants as instrumental variables (IVs) for the exposure [22]. Our two-sample MR study was conducted based on summary statistics from a genome-wide association study (GWAS) of 2634 LADA cases and 5947 controls [23], as well as a GWAS of 26,676 type 2 diabetes cases and 132,532 controls [24]. The IVs included 250 sentinel SNPs (independent SNPs) associated with smoking initiation (electronic supplementary material [ESM] Table 1) in a GWAS of up to 1.2 million European individuals [25]. Further details on the GWAS and MR methods can be found in the ESM Methods: Two-sample MR study; GWAS of LADA; GWAS of type 2 diabetes; and Genetic instruments for smoking.

### Statistical analysis

Differences in baseline characteristics were evaluated using two-sided *p* values, calculated by Student’s *t* test for means (±SD) of normally distributed variables (normality was assessed by visual inspection of distribution plots), Kruskal–Wallis test for medians (IQR) of non-normally distributed variables and *χ*^2^ test for proportions.

Conditional logistic regression estimated ORs with 95% CIs of LADA/type 2 diabetes in relation to smoking, snus use and total tobacco use in case–control data. Corresponding HRs were derived by Cox regression in HUNT, where study participants were followed from age at baseline until age at end of follow-up (HUNT2, 3 or 4), diabetes diagnosis, emigration or death. Models were adjusted for age and sex (matching variables in the logistic regression; age as underlying time scale in the Cox regression) (Model 1) together with BMI, educational level and alcohol consumption (Model 2). Exposures and covariates were updated at each new follow-up in HUNT, if possible. Furthermore, snus analyses were adjusted for smoking (never/former/current). Additional adjustment for physical activity and family history of diabetes did not change the effect estimates (<10% change in HR/OR) and were not retained in the final models. Pooled relative risks for smoking (RR_pooled_) were estimated through the inverse-variance weighted (IVW) method [26].

Interaction was defined as departure from additivity of effects and estimated as attributable proportion (AP) due to interaction with 95% CI. AP captures how much of the disease in the doubly exposed that can be attributed to the interaction and was calculated by the formula: ([RR_11_ – RR_10_ – RR_01_ + 1] / RR_11_) [27], where RR_11_ is risk in doubly exposed, RR_10_ is risk in non-tobacco users with low/intermediate risk genotypes, and RR_01_ is risk in non-tobacco users with high-risk genotypes. The reference group (RR_00_) comprised non-tobacco users with low/intermediate risk genotypes.

To address underlying mechanisms linking tobacco use to diabetes risk, we used multivariable linear regression to estimate differences in log_*e*_ transformed HOMA-IR and HOMA-B in relation to tobacco use in LADA/type 2 diabetes. Corresponding analyses of GADA were modelled using Tobit regression, to account for censoring of GADA. The analyses were based on case–control data, where HOMA and GADA were assessed at time of diagnosis.

Sensitivity analyses were performed to assess the validity of the genetic controls by re-running the main analyses with the incidence-density sampled controls. We assessed the association between smoking and LADA/type 2 diabetes separately in ESTRID and HUNT, and separately for each HUNT baseline (HUNT1, 2 or 3). Smoking and interaction with HLA genotypes was also assessed separately in ESTRID and HUNT.

We used the IVW method [28] to assess the potential causal link between smoking initiation and LADA/type 2 diabetes in the MR study, supplemented by other MR estimators and three conservative analyses excluding some SNPs (see ESM Methods: Data harmonisation and statistical analysis).

The observational analyses were performed in SAS 9.4 (SAS Institute, Cary, NC, USA) and the MR analyses were performed using the *MendelianRandomization* and MR-PRESSO (MR pleiotropy residual sum and outlier approach) package in R 4.0.4 [29]. All statistical tests were two-sided, with *p*<0.05 indicating statistical significance. Analysis codes are shared through GitHub (https://github.com/jeseds/Smoking-use-of-smokeless-tobacco-HLA-genotypes-and-incidence-of-LADA).

## Results

There were 838 individuals with LADA, 5764 with type 2 diabetes, 3036 controls (ESTRID) and 1,696,503 person-years of follow-up (HUNT). Compared with individuals with type 2 diabetes, those with LADA were less insulin resistant, had worse beta cell function as assessed by HOMA and lower levels of C-peptide, and were more likely to be treated with insulin (Table 1). A higher prevalence of high-risk HLA genotypes was seen in LADA than in type 2 diabetes and these genotypes conferred an RR_pooled_ (95% CI) of 2.62 (2.16, 3.18) for LADA and 0.93 (0.87, 1.00) for type 2 diabetes. All estimates hereafter refer to the fully adjusted model (Model 2).

**Table 1:** Characteristics of the study participants

Characteristic	ESTRID				HUNT			
	Controls	LADA	Type 2 diabetes	*p* value	No diabetes	LADA	Type 2 diabetes	*p* value
Individuals, *n*	3036	593	2038	−	74,326	245	3726	−
Men, %	27.8	53.0	60.0	0.002	47.9	47.8	53.2	0.10
Age at diagnosis, years^a^	56.1±10.3	59.1±12.3	63.2±10.4	<0.001	−	59.1±11.5	60.3±11.0	0.10
Age at baseline, years	−	−	−	−	56.2±17.3	52.9±11.7	54.7±11.5	0.01
BMI, kg/m^2^	25.4±4.1	28.5±5.6	31.2±5.4	<0.001	26.5±4.2	29.5±4.9	30.1±4.6	0.04
Using insulin, %^b^	−	39.9	5.9	<0.001	−	41.0	13.3	<0.001
HLA high-risk, %^c^	33.6	60.5	31.3	<0.001	29.0	48.9	27.3	<0.001
C-peptide, nmol/l	−	0.72 (0.45, 1.20)	1.20 (0.97, 1.60)	<0.001	−	0.59 (0.22, 0.99)	0.90 (0.63, 1.23)	<0.001
HOMA-IR	−	2.79 (1.82, 4.44)	3.56 (2.73, 4.77)	<0.001	−	2.0 (1.1, 2.7)	2.1 (1.5, 3.0)	0.08
HOMA-B	−	40.6 (15.0, 69.5)	71.1 (43.9, 95.9)	<0.001	−	56.2 (36.5, 79.2)	58.3 (38.4, 84.0)	0.39

Data are shown as mean±SD or median (IQR) unless stated otherwise

Clinical information (C-peptide) was available for 98% of the participants in ESTRID (LADA *n*=583, type 2 diabetes *n*=1990) and HOMA was available for 85% (LADA *n*=476, type 2 diabetes *n*=1752). C-peptide and HOMA measurements not from time of diagnosis were available for participants in HUNT1–3 (C-peptide, LADA *n*=122, type 2 diabetes *n*=1434 [39%]; HOMA, LADA *n*=80, type 2 diabetes *n*=1005 [27%]). Genetic information was available for 64% of the participants in ESTRID (LADA *n*=402, type 2 diabetes *n*=1289) and 92% of the participants in HUNT (LADA *n*=219, type 2 diabetes *n*=3421)

The *p* value is shown for LADA vs type 2 diabetes

^a^Age at participation for controls

^b^Current use of insulin

^c^High-risk genotypes were defined as carriers of *DR3/3*, *DR3/4*, *DR4/4*, or haplotypes of *DR4-DQ8* or *DR3-DQ2* (ESTRID) or carriers of at least one of the risk variants inferring either *DR3-DQ2* or *DR4-DQ8* (HUNT)

### Tobacco use and LADA

The risk of LADA was increased in current smokers (RR_pooled_ 1.30 [95% CI 1.06, 1.59]) and even more so in current, heavy smokers (RR_pooled_ 1.54 [95% CI 1.1, 2.14] for ≥20 cigarettes per day) but not in former smokers (Table 2). For snus, OR was estimated at 1.29 (95% CI 0.93, 1.80) in current users and 1.16 (95% CI 0.75, 1.79) in former users (Table 3). An almost doubled OR of 1.97 (95% CI 1.20, 3.24) was seen for ≥15 box-years vs never use, but no increased risk was seen in those with <15 box-years (Table 3). The association between LADA and ever smoking/snus use was weak (Tables 2, 3). The combination of current smoking and snus use vs never use was associated with a 2.46-fold (95% CI 1.50, 4.03) increase in the risk of LADA (Table 3).

**Table 2:** Pooled relative risks with 95% CIs for LADA and type 2 diabetes in relation to smoking

Smoking habit	LADA					Type 2 diabetes				
	Cases (*n*)	Controls (*n*)	Person-years	Model 1^a^ RR (95% CI)	Model 2^b^ RR (95% CI)	Cases (*n*)	Controls (*n*)	Person-years	Model 1^a^ RR (95% CI)	Model 2^b^ RR (95% CI)
Smoking										
Never	378	1467	729,291	1	1	2281	1467	729,291	1	1
Former	269	964	465,674	1.07 (0.89, 1.27)	1.08 (0.90, 1.29)	2084	964	465,674	1.12 (1.05, 1.20)	1.07 (1.00, 1.14)
Current	191	605	501,537	1.14 (0.94, 1.38)	1.30 (1.06, 1.59)	1399	605	501,537	1.11 (1.03, 1.20)	1.38 (1.28, 1.49)
Ever	460	1569	967,211	1.09 (0.94, 1.27)	1.16 (0.99, 1.36)	3483	1569	967,211	1.12 (1.05, 1.18)	1.20 (1.13, 1.28)
Intensity (current)										
No current smoking	647	2431	1,194,965	1	1	4365	2431	1,194,965	1	1
<20 cigarettes/day	127	497	358,396	1.00 (0.81, 1.25)	1.16 (0.93, 1.46)	920	497	358,396	1.03 (0.95, 1.12)	1.35 (1.24, 1.48)
≥20 cigarettes/day	55	98	112,955	1.54 (1.12, 2.11)	1.54 (1.11, 2.14)	425	98	112,955	1.35 (1.20, 1.50)	1.47 (1.31, 1.65)
Per 5 cigarettes	838	3036	1,696,503	1.06 (1.00, 1.14)	1.08 (1.01, 1.15)	5764	3036	1,696,503	1.03 (1.00, 1.06)	1.08 (1.05, 1.10)
Pack-years (ever)										
Never	378	1467	729,291	1	1	2281	1467	729,291	1	1
<15 pack-years	229	932	527,176	1.00 (0.84, 1.20)	1.11 (0.92, 1.33)	1463	932	527,176	0.99 (0.92, 1.07)	1.12 (1.03, 1.20)
≥15 pack-years	210	637	276,814	1.26 (1.03, 1.53)	1.28 (1.04, 1.57)	1660	637	276,814	1.39 (1.29, 1.50)	1.46 (1.34, 1.58)
Per 5 pack-years	838	3036	1,696,503	1.03 (1.00, 1.07)	1.03 (1.00, 1.06)	5764	3036	1,696,503	1.06 (1.05, 1.08)	1.05 (1.04, 1.07)

^a^Model 1 adjusted for age and sex

^b^Model 2 adjusted for age, sex, BMI, educational level and alcohol consumption

**Table 3:** ORs with 95% CIs for the association between snus use and LADA and type 2 diabetes

Snus use	LADA				Type 2 diabetes			
	Cases (*n*)	Controls (*n*)	Model 1^a^ OR (95% CI)	Model 2^b^ OR (95% CI)	Cases (*n*)	Controls (*n*)	Model 1^a^ OR (95% CI)	Model 2^b^ OR (95% CI)
Snus use								
Never	484	2684	1	1	1627	2684	1	1
Former	35	124	1.08 (0.71, 1.65)	1.16 (0.75, 1.79)	157	124	1.28 (0.97, 1.68)	1.20 (0.86, 1.67)
Current	74	228	1.19 (0.87, 1.64)	1.29 (0.93, 1.80)	254	228	1.42 (1.14, 1.77)	1.56 (1.20, 2.04)
Ever	109	352	1.15 (0.88, 1.51)	1.24 (0.94, 1.65)	411	352	1.37 (1.14, 1.64)	1.42 (1.14, 1.77)
Intensity (current)								
None	519	2808	1	1	1784	2808	1	1
<7 boxes per week	59	208	1.02 (0.73, 1.43)	1.15 (0.81, 1.63)	211	208	1.23 (0.98, 1.55)	1.45 (1.10, 1.91)
≥7 boxes per week	10	17	2.15 (0.92, 4.99)	1.49 (0.59, 3.80)	33	17	2.60 (1.37, 4.92)	1.85 (0.88, 3.89)
Box-years (ever)								
Never	484	2684	1	1	1627	2684	1	1
<15 box-years	73	293	0.98 (0.72, 1.32)	1.08 (0.78, 1.48)	292	293	1.21 (0.98, 1.48)	1.30 (1.02, 1.66)
≥15 box-years	36	59	1.94 (1.22, 3.10)	1.97 (1.20, 3.24)	119	59	2.08 (1.47, 2.94)	1.92 (1.27, 2.90)
Per 5 box-years	593	3036	1.11 (1.02, 1.22)	1.11 (1.01, 1.22)	2038	3036	1.15 (1.08, 1.23)	1.12 (1.04, 1.21)
Tobacco use								
Never	246	1379	1	1	700	1379	1	1
Former	171	891	1.07 (0.85, 1.34)	1.11 (0.88, 1.42)	766	891	1.38 (1.18, 1.60)	1.52 (1.26, 1.83)
Current	176	766	1.24 (0.98, 1.56)	1.37 (1.08, 1.75)	572	766	1.40 (1.19, 1.65)	1.78 (1.46, 2.18)
Ever	347	1657	1.15 (0.94, 1.39)	1.23 (1.00, 1.50)	1332	1657	1.39 (1.22, 1.58)	1.63 (1.39, 1.92)
Smoking/snus use								
None/none	417	2270	1	1	1466	2270	1	1
Current/none	102	538	1.20 (0.93, 1.54)	1.30 (1.00, 1.69)	318	538	1.12 (0.94, 1.33)	1.39 (1.13, 1.72)
None/current	40	161	0.87 (0.58, 1.29)	0.96 (0.63, 1.45)	166	161	1.18 (0.91, 1.53)	1.48 (1.08, 2.03)
Current/current	34	67	2.28 (1.42, 3.65)	2.46 (1.50, 4.03)	88	67	2.04 (1.42, 2.95)	1.98 (1.30, 3.04)

Data for snus use were from case−control (ESTRID) only

^a^Model 1 adjusted for age and sex

^b^Model 2 adjusted for age, sex, BMI, educational level and alcohol consumption; snus analyses additionally adjusted for smoking

### Tobacco use and type 2 diabetes

Current and former vs never smoking showed RR_pooled_ 1.38 (95% CI 1.28, 1.49) and RR_pooled_ 1.07 (95% CI 1.00, 1.14), respectively, in type 2 diabetes, and the risk increased with number of pack-years (Table 2). Type 2 diabetes was also associated with current and ever, but not former, snus use (Table 3). The combination of current smoking and snus use was associated with a 1.98-fold (95% CI 1.30, 3.04) increase in the risk of type 2 diabetes (Table 3).

### Tobacco use, HLA high-risk genotypes and LADA

There was additive interaction between current smoking and high-risk HLA genotypes with RR_pooled_ in those doubly exposed estimated at 3.60 (95% CI 2.59, 5.00), and an AP due to interaction of 0.27 (95% CI 0.01, 0.53) (Table 4). Under the assumption of causality, this implies that 27% of the doubly exposed cases are attributable to interaction between smoking and high-risk HLA genotypes. Similarly, snus users with high genetic risk had an OR of 6.65 (95% CI 3.31, 13.36), with a non-significant AP estimated at 0.42 (95% CI −0.01, 0.85) (ESM Table 2). Results from the corresponding analyses for tobacco use and HLA are presented in ESM Table 3.

**Table 4:** Pooled RRs with 95% CIs for combinations of smoking and HLA genotypes in the risk of LADA, and pooled APs due to interaction with 95% CI

Smoking	HLA genotype	Cases (*n*)	Controls (*n*)	Person-years	Model 1^a^ RR (95% CI)	Model 2^b^ RR (95% CI)	AP (95% CI)
Ever smoking	High-risk						
−	−	120	285	425,079	1	1	
+	−	151	317	574,688	1.01 (0.77, 1.34)	1.07 (0.81, 1.42)	
−	+	156	137	177,868	2.52 (1.88, 3.39)	2.52 (1.86, 3.41)	
+	+	194	167	228,292	2.74 (2.09, 3.60)	3.10 (2.34, 4.10)	
Pooled							0.19 (−0.06, 0.44)
Current smoking							
−	−	208	488	705,267	1	1	
+	−	63	114	294,500	1.01 (0.73, 1.41)	1.15 (0.82, 1.60)	
−	+	270	247	291,593	2.55 (2.04, 3.17)	2.59 (2.06, 3.24)	
+	+	80	57	114,567	2.88 (2.10, 3.97)	3.60 (2.59, 5.00)	
Pooled							0.27 (0.01, 0.53)
≥15 pack-years							
−	−	204	474	815,808	1	1	
+	−	67	128	183,959	1.24 (0.89, 1.75)	1.19 (0.84, 1.69)	
−	+	259	234	334,265	2.71 (2.16, 3.40)	2.73 (2.17, 3.43)	
+	+	91	70	71,896	3.29 (2.38, 4.56)	3.57 (2.55, 4.99)	
Pooled							0.19 (−0.10, 0.48)

^a^Model 1 adjusted for age and sex

^b^Model 2 adjusted for age, sex, BMI, educational level and alcohol consumption

### Tobacco use and HOMA-IR, HOMA-B and GADA

Smoking was positively associated with HOMA-IR and HOMA-B in both LADA and type 2 diabetes (ESM Table 4). In LADA, every five pack-years were associated with a 2.8% higher HOMA-IR (*β*=0.0276, *p*=0.0488). There was no significant association between tobacco use and levels of GADA.

### Sensitivity analyses

Study-specific analyses revealed a positive association between smoking and LADA in the Swedish but not the Norwegian study, while the association with type 2 diabetes was seen in both datasets (ESM Tables 5, 6). The associations remained when restricted to LADA with high (above median) GADA levels (RR_pooled_ 1.45 (95% CI 1.12, 1.89) for current smoking). Separate analyses by HUNT baseline revealed that smoking was associated with a reduced risk of LADA in the first wave (HUNT1) and an increased risk in later waves (ESM Table 7). For the combination of smoking and HLA high-risk genotypes, study-specific APs were compatible with interaction but were not statistically significant (ESM Tables 8, 9). The association between smoking and LADA/type 2 diabetes was similar when we used controls collected within ESTRID instead of genetic controls (ESM Table 10). Since snus use was more prevalent in men, we restricted the analysis to men and found similar results (ESM Table 11). Restricting the analysis to never-smokers revealed similar associations primarily for type 2 diabetes (ESM Table 12).

### MR analyses of smoking and LADA/type 2 diabetes

**Fig. 1:**
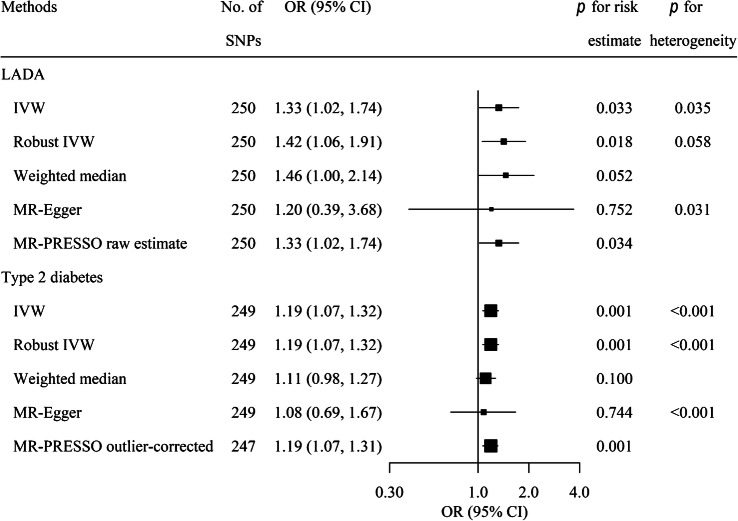
MR analysis on associations of smoking with LADA and type 2 diabetes. MR-Egger (Egger regression of MR): *I*^2^_GX_=0.303, intercept=0.002 and *p* for directional pleiotropy=0.848 for LADA; *I*^2^_GX_=0.321, intercept=0.002 and *p* for directional pleiotropy=0.647 for type 2 diabetes. *I*^2^_GX_ is used to quantify the strength of NOME (NO Measurement Error) violation for a set of instruments used for MR-Egger regression. An *I*^2^_GX_ much less than 1 indicates bias in the MR-Egger causal estimate. MR-PRESSO detected no outliers for the association between smoking and LADA and detected rs1109480 and rs8005334 as outliers for smoking and type 2 diabetes. The outliers were excluded from the outlier corrected estimate (*p* for distortion of estimate: 0.953). The sizes of the squares are proportional to the inverse variance of risk estimates. One of the 250 SNPs was unavailable in the GWAS dataset of type 2 diabetes and therefore only 249 SNPs were used when assessing the association of smoking with type 2 diabetes

Genetic predisposition to smoking initiation was associated with a higher risk of LADA (OR 1.33 per unit [on the log odds scale] increase in the risk of smoking [95% CI 1.02, 1.74]) and type 2 diabetes (OR 1.19 [95% CI 1.07, 1.32]) according to the IVW method (Fig. 1). We observed no strong evidence of asymmetry in the funnel plots (ESM Figs 1, 2). The same direction of association was obtained using other MR estimators (Fig. 1), and in the three conservative analyses (ESM Table 13).

## Discussion

### Main findings

We observed an increased risk of LADA in smokers and snus users, and confirmed that tobacco users are at increased risk of type 2 diabetes [1]. The MR analyses provided genetic support for a causal link between smoking initiation and LADA. Furthermore, there was an indication of interaction between HLA high-risk genotypes and smoking in relation to LADA, suggesting that genetic susceptibility may accentuate the adverse effects of smoking on the risk of LADA.

### Main findings in relation to previous studies

Previous observations regarding smoking and the risk of LADA are limited [8, 9] and are based partly on the same Scandinavian data as this study but with fewer cases and shorter follow-up time (ESTRID data collected 2010–2014 instead of 2010–2019 and HUNT data for the period 1984–2008 instead of 1984–2019). These results pointed to an increased risk in smokers in the Swedish data but a decreased risk in the Norwegian cohort. Small numbers may have contributed to these contradictory results. Furthermore, both protective effects of smoking on LADA (such as suppression of autoimmunity [5]) and adverse effects (such as promotion of insulin resistance [3]) seem possible, and the net effect may vary by population characteristics, including genetic susceptibility. In this study, the positive association between smoking and LADA was primarily seen in the Swedish data, whereas interaction with HLA genotypes, although not significant, appeared similar in both datasets. The MR results supported the notion of smoking having primarily adverse effects on LADA risk. With regard to type 2 diabetes, the results indicated adverse effects of smoking, replicating a previous MR observation [2]. Of note, twin studies have found that genetic factors influence smoking behaviours and, furthermore, that genes promoting nicotine dependence are distinct from those promoting diabetes (30). We found a positive association between smoking and HOMA-IR in LADA as well as in type 2 diabetes, whereas there was no significant association with levels of GADA. This suggests that increasing insulin resistance, and not direct effects on autoimmunity, may be the main driver of the excess risk of LADA seen in tobacco users, and that smoking serves as a promotor rather than a trigger in the aetiology of LADA. Nicotine has previously been shown to promote insulin resistance [3] and the similar risks seen in smokers and snus users support nicotine as the main component of tobacco products driving these associations. However, our findings may be at odds with experimental studies showing a protective effect of nicotine on autoreactivity and beta cell survival [30, 31].

We observed interaction between smoking and HLA high-risk genotypes. Results were similar but not statistically significant for snus use and HLA. These findings are in line with previous studies in rheumatoid arthritis [10] and multiple sclerosis [11]. A potential explanation for the observed interaction in relation to LADA is that insulin resistance speeds up the progression to manifest diabetes in individuals with an intrinsically high rate of beta cell apoptosis [32], and this process may be more pronounced in high-risk HLA carriers due to abnormal immune responses to environmental factors [7]. However, since this is the first study investigating a potential interaction between tobacco use and HLA genotypes in relation to autoimmune diabetes, confirmations are clearly warranted.

### Strengths and limitations

Strengths include the large number of incident LADA cases, the use of two study populations, the population-based design, and detailed information on tobacco use, potential confounders, clinical and genetic factors. In addition, we performed an MR study designed to minimise confounding bias and reverse causation, since the genetic instruments are randomly assigned from parents and determined before the occurrence of outcomes [33].

Self-reported information on tobacco use is a limitation although it is correlated with blood cotinine levels [34]. The prospective design of the HUNT study indicates that any smoking misclassification is non-differential, which will lead to diluted associations. Recall bias is a concern in the ESTRID study, as individuals may exaggerate or under-report their smoking habits because of their diagnosis. However, time between diagnosis and reporting was relatively short. Moreover, our results regarding smoking and type 2 diabetes were in line with previous findings based on prospective studies [1], supporting the validity of our data.

The observational association between smoking and diabetes was confirmed by the MR results. The validity of these results relies on several assumptions [35] and it is crucial that an IV only affects the outcome through the exposure, not through a direct pathway to the outcome or via a confounder. The MR assumptions are difficult to test; however, the positive association between smoking and LADA/type 2 diabetes remained in conservative analyses that excluded some potential pleiotropic SNPs. Excluding these SNPs does, however, not necessarily mean that the SNPs indeed have pleiotropic effects that would break the MR assumption. Finally, there can be ‘non-compliance’ in MR analyses due to epigenetic modification of SNPs for smoking [36]. Still, such non-compliance will most likely attenuate the observed association. For further discussion about MR results, see ESM Text.

We used the presence of GADA as a criterion for LADA but other autoantibodies may be present and individuals with such positivity would be classified as having type 2 diabetes. However, GADA is present in >90% of individuals with LADA [37] and typically persist over time, although with declining frequency [38]. The inability to separate LADA from adult-onset type 1 diabetes in HUNT could lead to some individuals with type 1 diabetes being misclassified as having LADA, possibly diluting the association between smoking and LADA. In this context it is noteworthy that the distinction between LADA and type 1 diabetes with adult onset is not clear-cut. The ADA proposes that LADA should be viewed as a subtype of type 1 diabetes [39].

The specificity of the GADA assay implies that some individuals with type 2 diabetes will be misclassified as having LADA. Importantly, smoking was associated with more autoimmune LADA, where potential misclassification of individuals with type 2 diabetes likely is minor. Finally, LADA is a heterogeneous disease [7], and whether these findings can be generalised to non-Scandinavian populations remains to be investigated.

In conclusion, this study based on the combination of observational and MR data indicates that smoking and snus use increase the risk of LADA and suggests that the excess risk conferred by smoking is augmented in genetically susceptible individuals. The association may be attributed to an insulin resistance promoting effect of nicotine. Our results suggest that cessation of tobacco use should be a priority, not only in individuals susceptible to type 2 diabetes but also in the prevention of LADA. However, larger studies, as well as studies in other ethnic populations, are clearly needed. The impact of potential interactions with other risk genotypes outside the HLA gene complex should be explored in relation to LADA.

## Supplementary Information

ESM (PDF 823 kb)

## Data Availability

The datasets analysed in the current study are available from the corresponding author upon reasonable request (ESTRID) and with permission of the HUNT study by applying to the HUNT study data access committee. The MR analysis used summary data and these data are publicly available. Analysis codes are shared through GitHub (https://github.com/jeseds/Smoking-use-of-smokeless-tobacco-HLA-genotypes-and-incidence-of-LADA).

## Abbreviations

ANDIS: All New Diabetics in Scania
AP: Attributable proportion due to interaction
EIRA: Epidemiological Investigation of Rheumatoid Arthritis
ESTRID: Epidemiological Study of Risk Factors for LADA and Type 2 Diabetes
GADA: GAD antibody
GWAS: Genome-wide association study
HUNT: The Trøndelag Health Study
IV: Instrumental variable
IVW: Inverse-variance weighted
LADA: Latent autoimmune diabetes in adults
MR: Mendelian randomisation
MR-PRESSO: MR pleiotropy residual sum and outlier approach
