# Supplementary material for: Smoking, use of smokeless tobacco, HLA genotypes and incidence of latent autoimmune diabetes in adults
Source: Diabetologia. 2022 Jul 28;66(1):70–81. doi: 10.1007/s00125-022-05763-w (PMC9729119; doi:10.1007/s00125-022-05763-w)
Supplement: Supplementary file 1 — (PDF 823 kb) [file 125_2022_5763_MOESM1_ESM.pdf]

Supplementary material

Smoking, use of smokeless tobacco, HLA genotypes, and incidence of latent autoimmune diabetes in adults

Jessica Edstorp[1], Yuxia Wei[1], Emma Ahlqvist[2], Lars Alfredsson[1,3], Valdemar Grill[4], Leif Groop[2,5], Bahareh Rasouli[1,6], Elin P Sørgjerd[7,8], Per M Thorsby[9,10], Tiinamaija Tuomi[2,5,11,12, 13], Bjørn O Åsvold[7,8,14] and Sofia Carlsson[1]

[1]Institute of Environmental Medicine, Karolinska Institutet, Stockholm, Sweden
[2]Department of Clinical Sciences in Malmö, Clinical Research Centre, Lund University, Malmö, Sweden
[3]Center for Occupational and Environmental Medicine, Region Stockholm, Stockholm, Sweden
[4]Department of Clinical and Molecular Medicine, Norwegian University of Science and Technology, Trondheim, Norway
[5]Institute for Molecular Medicine Finland, Helsinki University, Helsinki, Finland
[6]Department of Global Health and Population, Harvard TH Chan School of Public Health, Boston, USA
[7]HUNT Research Centre, Department of Public Health and Nursing, NTNU, Norwegian University of Science and Technology, Trondheim, Norway
[8]Department of Endocrinology, Clinic of Medicine, St Olavs Hospital, Trondheim, Norway
[9]Hormone Laboratory, Department of Medical Biochemistry, Oslo University Hospital, Aker, Oslo, Norway
[10]Biochemical endocrinology and metabolism research group, Oslo University Hospital, Aker, Oslo, Norway
[11]Division of Endocrinology, Abdominal Center, Helsinki University Hospital, Helsinki, Finland
[12]Research Program for Diabetes and Obesity, University of Helsinki, Helsinki, Finland
[13]Folkhälsan Research Center, Helsinki, Finland
[14]K.G. Jebsen Center for Genetic Epidemiology, Department of Public Health and Nursing, NTNU, Norwegian University of Science and Technology, Trondheim, Norway

ESM Methods

Two-sample Mendelian randomisation (MR) study

A typical two-sample MR study uses uncorrelated genetic variants as instrumental variables to proxy modifiable exposures and provides a novel opportunity for causal inference [1, 2]. Such a study is based on summary statistics (β and standard errors) for the associations between genetic instruments and the exposure, and between genetic instruments and the outcome [3] from genome-wide association studies (GWAS).

Valid genetic instruments in MR studies affect the outcome only through the exposure, and the genetic instruments should not affect the outcome directly or through confounders of the exposure-outcome association [4].

GWAS of LADA

There is only one GWAS of LADA hitherto [7], which was conducted in 2634 LADA cases versus 5947 population controls of European ancestry. LADA was defined according to age of diabetes onset, presence of diabetes-related autoimmune antibodies and the lack of insulin dependence within the first six months or one year of diagnosis. Summary statistics for the association between the 250 SNPs and LADA were extracted from this GWAS.

GWAS of type 2 diabetes

The DIAbetes Genetics Replication And Meta-analysis Consortium (DIAGRAM) conducted a GWAS of type 2 diabetes including 26,676 cases and 132,532 controls of European ancestry [8]. Summary statistics for the association between SNPs and type 2 diabetes were extracted from this GWAS. One of the 250 SNPs was unavailable in this GWAS and only 249 SNPs were used as instrumental variables for smoking when assessing its association with type 2 diabetes.

Genetic instruments for smoking

The GWAS and Sequencing Consortium of Alcohol and Nicotine use (GSCAN) conducted a GWAS in up to 1.2 million European individuals and identified 259 sentinel single nucleotide polymorphisms (SNPs) for smoking initiation (ever smoking) [5]. A total of 250 out of the 259 SNPs were available in the GWAS of LADA (described below) and were used as instrumental variables for smoking in this MR study. These SNPs explained approximately 4% of the variance in smoking. Three (rs6011779, rs11783093, and rs12027999) of the 250 SNPs are closely related to CHRN genes, which encode subunits of neuronal nicotinic acetylcholine receptors (nAChRs) [6]. There was no SNP located near or in linkage disequilibrium (LD) with genes of the human leucocyte antigen (HLA) among the 250 SNPs used as instrumental variables. Summary statistics for the marginal associations between the 250 SNPs and smoking were extracted from the GWAS dataset generated by GSCAN based on 632,802 individuals (the 23andMe cohort was excluded from the released GWAS dataset).

Data harmonisation

All SNPs were oriented, with the smoking-increasing alleles as effect alleles.

Statistical analysis

We used the inverse-variance weighted (IVW) method as the main method to estimate the association between smoking and LADA as well as type 2 diabetes. This method assumes that all genetic instruments are valid and is the most accurate when this assumption is satisfied [4]. We also used other MR estimators, which did not require all the SNPs to be valid, as a supplement. Other MR estimators included the robust IVW, weighted median, the Egger regression of Mendelian randomisation (MR-Egger), and the Mendelian randomisation pleiotropy residual sum and outlier approach (MR-PRESSO) methods. The robust IVW replaces the standard linear regression model in IVW with the robust regression model [9]. The weighted median method can give reliable estimates when more than half of the genetic instruments are valid [10]. We constructed funnel plots for a visual inspection of asymmetry and performed formal statistical tests for directional pleiotropy using MR-Egger and MR-PRESSO. The MR-Egger [11] and MR-PRESSO [12] methods can help detect and correct for potential pleiotropy, but the MR-Egger method often suffers from low statistical power.

We also did three conservative analyses by excluding some SNPs from the 250 genetic instruments, to minimise the possibility that genetic instruments affect the outcome directly or through potential confounders. The first conservative analysis excluded SNPs associated with any trait (except smoking itself) at $p<5\times10^{-8}$. The second conservative analysis further excluded SNPs associated with diabetes-related traits at $p<5\times10^{-4}$ (0.05/250). The third conservative analysis additionally excluded SNPs associated with alcohol-related traits at $p<5\times10^{-4}$ (0.05/250). The associations between SNPs and such traits were identified from Phenoscanner, which is a database holding publicly available results from large-scale GWAS and currently contains over 65 billion associations and over 150 million unique genetic variants [13, 14].

ESM Discussion about MR results

Causal inferences are more plausible when there are consistent findings across different MR methods. In our study, different MR estimators (except for MR-Egger) estimated the same direction and similar magnitude of association for LADA as well as type 2 diabetes, indicating the robustness of our findings. I^2_{GX} statistics in the MR-Egger analysis were significantly less than 1, meaning that the MR-Egger causal effect estimates were biased, as reflected by the much smaller ORs estimated by MR-Egger than ORs estimated by other MR estimators. Moreover, MR-Egger often suffers from low statistical power, which was reflected by the wider 95% CIs estimated from MR-Egger than 95% CIs estimated by other MR estimators (**Figure 1**). A low I^2_{GX} statistic means that either the NOME (NO Measurement Error) assumption or the InSIDE (Instrument Strength Independent of Direct Effect) assumption that MR-Egger relies on is violated [15]. The violation of NOME assumption indicates "weak instrument bias", which will bias the risk estimate to null in MR-Egger analysis [15]. "Weak instrument bias" can also be a problem for other MR estimators such as IVW. However, different from MR-Egger, the strength of instruments in other MR estimators relies on F statistics and not on I^2_{GX}. An F statistic of >10 is generally regarded as evidence of strong instrument strength [2] and the F statistics for 249 out of the 250 SNPs used in the present MR analysis were >30 (ESM Table 1). The InSIDE assumption is violated if pleiotropic effects act via a confounder [11]. In addition to MR-Egger, other MR methods such as MR-PRESSO also need to satisfy the InSIDE assumption [12]. However, we have excluded SNPs associated with any other trait at genome-wide significance level from conservative analysis 1 and positive associations of smoking with LADA and type 2 diabetes still existed.

The *p* values for heterogeneity tests in different MR estimators indicated the presence of heterogeneity. The existence of heterogeneity, however, does not necessarily imply the existence of pleiotropy. Heterogeneity can also be due to different biological mechanisms linking different SNPs to the exposure and there is no violation of MR assumptions in this scenario [3]. Heterogeneity can also exist when the overall pleiotropic effects from different SNPs happen to cancel out ("balanced pleiotropy") [16]. The causal estimates from different MR methods are still unbiased in the case of balanced pleiotropy. The outlier test for MR-PRESSO in fact detected no outlier for the smoking-LADA association (**Figure 1**). MR-PRESSO detected two outliers for the association between smoking and type 2 diabetes, but the outlier-corrected estimates are similar to the ORs estimated by IVW. Therefore, horizontal pleiotropy does not seem to severely bias the causal estimates in our study.

ESM Table 1. Basic information about the 250 SNPs used in the Mendelian randomisation analysis.

SNP	Effect allele	Other allele	SNP-smoking association				SNP-LADA association		
			BETA	SE	F statistics	*P*	BETA	SE	*P*
rs10042827	C	T	0.0187	0.0038	24.2	8.63E-07	-0.0024	0.0400	0.952
rs1004787	A	G	0.0299	0.0036	70.2	5.27E-17	0.0270	0.0373	0.472
rs1008078	T	C	0.0222	0.0036	37.6	8.61E-10	0.0029	0.0376	0.939
rs10233018	G	A	0.0271	0.0036	57.9	2.75E-14	0.0152	0.0373	0.686
rs10279261	G	A	0.0214	0.0037	34.2	5.00E-09	0.0204	0.0388	0.601
rs10446419	A	G	0.0187	0.0044	18.2	1.96E-05	-0.0603	0.0464	0.196
rs10490159	T	C	0.0216	0.0036	35.5	2.59E-09	-0.0367	0.0382	0.340
rs1050847	C	T	0.0216	0.0036	36.3	1.67E-09	-0.0060	0.0378	0.875
rs1059490	T	C	0.0169	0.0037	20.7	5.26E-06	-0.0625	0.0399	0.120
rs10789369	A	G	0.0244	0.0037	44.0	3.23E-11	-0.1090	0.0381	0.004
rs10858334	G	C	0.0242	0.0053	20.5	5.84E-06	0.0035	0.0565	0.951
rs10873871	G	A	0.0207	0.0043	22.7	1.90E-06	0.0421	0.0452	0.354
rs10885480	T	C	0.0162	0.0039	17.1	3.53E-05	0.0719	0.0414	0.084
rs10905461	T	C	0.0240	0.0041	33.4	7.35E-09	-0.0390	0.0439	0.376
rs10914684	G	A	0.0183	0.0038	23.0	1.58E-06	-0.0561	0.0400	0.163
rs10935779	C	T	0.0146	0.0036	16.5	4.91E-05	-0.0360	0.0379	0.346
rs10945141	A	G	0.0222	0.0041	30.1	4.18E-08	-0.0362	0.0422	0.394
rs10953957	A	G	0.0185	0.0037	25.4	4.72E-07	-0.0189	0.0383	0.624
rs10966092	T	C	0.0174	0.0040	18.8	1.43E-05	-0.0747	0.0420	0.077
rs10969352	A	T	0.0164	0.0036	21.4	3.67E-06	0.0410	0.0371	0.272
rs11057005	A	G	0.0209	0.0036	34.2	4.85E-09	0.0563	0.0375	0.136
rs11076962	C	T	0.0189	0.0040	22.7	1.94E-06	0.0879	0.0407	0.032
rs1109480	G	A	0.0188	0.0037	26.5	2.64E-07	-0.0029	0.0381	0.941
rs11128203	A	T	0.0177	0.0036	24.8	6.39E-07	-0.0010	0.0373	0.979
rs11162019	C	T	0.0205	0.0037	30.7	3.09E-08	0.0283	0.0384	0.464
rs1116690	G	A	0.0161	0.0041	15.6	7.82E-05	0.0216	0.0429	0.617
rs11258417	C	T	0.0160	0.0036	19.5	9.83E-06	0.0688	0.0383	0.074
rs1126757	T	C	0.0187	0.0036	27.6	1.53E-07	0.0448	0.0374	0.234
rs112725451	T	C	0.0271	0.0048	32.2	1.38E-08	0.0509	0.0481	0.293
rs113230003	G	A	0.0206	0.0041	25.2	5.19E-07	0.0566	0.0447	0.208
rs1139897	G	A	0.0246	0.0042	34.6	4.08E-09	-0.0208	0.0431	0.631
rs1150668	T	G	0.0181	0.0036	25.1	5.55E-07	0.0244	0.0410	0.555
rs1160685	G	C	0.0208	0.0036	33.5	7.20E-09	-0.0437	0.0374	0.245
rs11611651	A	G	0.0339	0.0063	28.8	8.02E-08	-0.0875	0.0675	0.198
rs11642231	G	A	0.0142	0.0036	15.1	1.04E-04	-0.0326	0.0381	0.394
rs11651955	G	A	0.0173	0.0036	23.7	1.14E-06	-0.0102	0.0373	0.785
rs11692435	A	G	0.0304	0.0062	24.2	8.71E-07	0.1248	0.0624	0.047
rs11713899	C	A	0.0210	0.0047	19.6	9.48E-06	0.0193	0.0502	0.702
rs1173461	T	C	0.0171	0.0038	20.3	6.49E-06	-0.0646	0.0405	0.112
rs11766326	T	C	0.0172	0.0037	21.2	4.13E-06	0.0065	0.0373	0.862
rs11768481	C	A	0.0232	0.0038	38.0	7.00E-10	-0.0153	0.0393	0.700

SNP	Effect allele	Other allele	SNP-smoking association				SNP-LADA association		
			BETA	SE	F statistics	*P*	BETA	SE	*P*
rs117734003	C	G	0.0290	0.0071	16.5	4.91E-05	0.1664	0.0749	0.027
rs11783093	C	T	0.0405	0.0049	67.9	1.74E-16	0.0540	0.0514	0.296
rs118202	G	T	0.0380	0.0045	70.2	5.27E-17	-0.0150	0.0468	0.749
rs11872397	G	A	0.0248	0.0041	36.6	1.43E-09	0.0401	0.0430	0.354
rs11956866	T	G	0.0146	0.0036	16.6	4.72E-05	-0.0424	0.0380	0.268
rs12022778	C	A	0.0259	0.0044	34.7	3.87E-09	0.0196	0.0460	0.672
rs12027999	T	C	0.0331	0.0053	38.4	5.76E-10	0.0185	0.0568	0.745
rs12195240	A	G	0.0274	0.0040	48.0	4.33E-12	-0.0078	0.0410	0.851
rs12244388	A	G	0.0284	0.0037	57.3	3.76E-14	0.0922	0.0387	0.018
rs12442563	G	T	0.0273	0.0043	40.9	1.62E-10	0.0346	0.0436	0.431
rs12474587	T	G	0.0276	0.0036	59.5	1.25E-14	-0.0667	0.0374	0.076
rs12517438	G	T	0.0189	0.0036	28.2	1.10E-07	0.0060	0.0372	0.872
rs12530388	A	C	0.0207	0.0036	33.8	6.13E-09	-0.0247	0.0372	0.509
rs12563365	A	G	0.0168	0.0036	21.9	2.83E-06	0.0157	0.0373	0.676
rs12714017	C	T	0.0194	0.0037	27.1	1.89E-07	0.0464	0.0373	0.216
rs12739243	T	C	0.0233	0.0043	29.5	5.56E-08	0.0687	0.0443	0.123
rs12755632	A	G	0.0158	0.0038	17.1	3.50E-05	0.0244	0.0396	0.540
rs12918191	A	G	0.0194	0.0042	21.7	3.11E-06	0.0196	0.0435	0.655
rs1291821	G	A	0.0159	0.0036	19.9	8.32E-06	0.0390	0.0374	0.300
rs13007361	A	G	0.0223	0.0045	25.1	5.45E-07	0.0736	0.0470	0.120
rs13066050	T	C	0.0158	0.0044	13.1	2.99E-04	-0.0555	0.0454	0.224
rs13109980	G	A	0.0244	0.0038	41.8	9.99E-11	0.0170	0.0397	0.670
rs13110073	T	C	0.0250	0.0036	47.0	7.25E-12	-0.0322	0.0382	0.402
rs13237637	G	C	0.0211	0.0036	35.2	3.00E-09	0.0215	0.0372	0.566
rs13261666	G	T	0.0269	0.0036	57.2	3.90E-14	-0.0137	0.0371	0.713
rs13392222	A	C	0.0221	0.0051	18.8	1.42E-05	-0.0151	0.0512	0.769
rs13437771	A	G	0.0293	0.0049	36.0	2.02E-09	-0.0738	0.0512	0.152
rs1373178	T	G	0.0198	0.0036	30.0	4.34E-08	-0.0081	0.0378	0.831
rs1381287	T	C	0.0188	0.0036	27.6	1.49E-07	-0.0487	0.0375	0.197
rs1381775	T	C	0.0149	0.0039	14.3	1.55E-04	-0.0163	0.0411	0.693
rs1385108	T	C	0.0247	0.0042	35.2	3.00E-09	0.0498	0.0440	0.261
rs13906	C	T	0.0208	0.0057	13.3	2.72E-04	0.0713	0.0593	0.232
rs139896	C	T	0.0141	0.0037	14.2	1.69E-04	0.0389	0.0390	0.320
rs1435672	C	T	0.0159	0.0036	19.8	8.55E-06	0.0371	0.0375	0.325
rs1445649	C	T	0.0240	0.0036	45.3	1.68E-11	0.0483	0.0372	0.197
rs1514176	G	A	0.0219	0.0036	36.9	1.25E-09	0.0454	0.0379	0.234
rs1518393	C	A	0.0205	0.0037	31.5	2.03E-08	-0.0047	0.0383	0.904
rs1549979	C	T	0.0309	0.0037	70.7	4.18E-17	0.0624	0.0385	0.107
rs1555445	T	A	0.0226	0.0038	34.8	3.65E-09	0.0160	0.0395	0.686
rs160631	T	G	0.0229	0.0040	32.6	1.11E-08	0.0086	0.0415	0.836
rs16826827	T	C	0.0198	0.0054	13.7	2.18E-04	-0.0094	0.0569	0.870
rs16828799	T	G	0.0215	0.0048	19.6	9.68E-06	-0.0255	0.0503	0.614
rs1714521	A	C	0.0195	0.0036	29.3	6.33E-08	0.0661	0.0381	0.085
rs17197663	G	A	0.0158	0.0053	8.8	3.09E-03	-0.0135	0.0578	0.817
rs17229285	C	T	0.0171	0.0036	23.2	1.47E-06	-0.0190	0.0374	0.614

SNP	Effect allele	Other allele	SNP-smoking association				SNP-LADA association		
			BETA	SE	F statistics	*P*	BETA	SE	*P*
rs1733760	C	T	0.0152	0.0036	18.3	1.90E-05	-0.0209	0.0374	0.579
rs1737329	G	C	0.0158	0.0040	15.4	8.69E-05	0.0029	0.0430	0.946
rs17554906	C	G	0.0132	0.0036	13.6	2.26E-04	0.0100	0.0374	0.790
rs1759433	A	G	0.0179	0.0036	25.2	5.10E-07	-0.0177	0.0370	0.635
rs17692129	T	C	0.0198	0.0038	27.8	1.32E-07	-0.0821	0.0400	0.042
rs1799068	T	G	0.0161	0.0037	19.4	1.05E-05	0.0469	0.0384	0.224
rs1811739	A	G	0.0173	0.0041	17.6	2.79E-05	-0.0710	0.0426	0.098
rs1889571	G	T	0.0247	0.0053	21.9	2.89E-06	0.0486	0.0544	0.374
rs1901477	G	A	0.0299	0.0037	64.5	9.84E-16	-0.0852	0.0369	0.022
rs1910236	A	G	0.0192	0.0036	29.2	6.44E-08	0.0184	0.0374	0.625
rs1927901	T	C	0.0149	0.0036	17.4	3.06E-05	-0.0241	0.0379	0.527
rs1930371	C	T	0.0175	0.0042	17.7	2.53E-05	0.0167	0.0445	0.709
rs1931431	C	G	0.0181	0.0036	25.8	3.73E-07	-0.0684	0.0371	0.067
rs1935571	T	G	0.0185	0.0036	27.0	2.05E-07	0.0296	0.0375	0.432
rs1937443	G	C	0.0261	0.0036	52.8	3.69E-13	-0.0392	0.0372	0.295
rs2010921	A	G	0.0187	0.0038	23.8	1.07E-06	-0.0286	0.0400	0.478
rs2145451	T	C	0.0232	0.0045	26.4	2.71E-07	0.0033	0.0479	0.945
rs2155646	C	T	0.0435	0.0036	143.0	4.94E-33	0.0144	0.0379	0.705
rs221988	A	C	0.0151	0.0037	17.2	3.43E-05	0.0153	0.0382	0.691
rs2276825	C	T	0.0232	0.0042	31.2	2.38E-08	0.0471	0.0428	0.273
rs2279829	C	T	0.0166	0.0043	14.9	1.14E-04	0.0205	0.0448	0.649
rs2289791	G	T	0.0187	0.0041	20.6	5.70E-06	-0.0584	0.0428	0.174
rs2344976	T	C	0.0206	0.0037	31.7	1.80E-08	0.0275	0.0382	0.473
rs2359180	A	G	0.0150	0.0039	14.6	1.30E-04	0.0195	0.0525	0.712
rs2378662	A	G	0.0209	0.0036	34.5	4.16E-09	0.0676	0.0372	0.071
rs238896	G	A	0.0171	0.0036	23.2	1.43E-06	0.0640	0.0377	0.091
rs2526390	T	C	0.0220	0.0038	34.1	5.19E-09	-0.0536	0.0397	0.180
rs2587507	T	C	0.0177	0.0036	24.8	6.21E-07	-0.0258	0.0376	0.495
rs2637869	A	G	0.0186	0.0039	23.2	1.46E-06	0.0283	0.0400	0.482
rs2710634	T	C	0.0182	0.0036	26.2	3.07E-07	0.0423	0.0371	0.256
rs2734390	G	A	0.0127	0.0037	11.8	5.89E-04	0.0062	0.0389	0.873
rs2796793	A	G	0.0135	0.0036	14.4	1.45E-04	-0.0061	0.0374	0.870
rs281296	A	G	0.0289	0.0037	60.5	7.54E-15	0.0650	0.0387	0.095
rs28441558	T	C	0.0324	0.0075	18.7	1.53E-05	-0.0636	0.0775	0.415
rs2901785	G	A	0.0156	0.0036	19.0	1.28E-05	0.0640	0.0374	0.088
rs290601	T	C	0.0143	0.0040	12.9	3.31E-04	-0.0252	0.0408	0.539
rs2939756	G	A	0.0177	0.0036	24.6	7.13E-07	-0.0173	0.0371	0.642
rs301807	G	A	0.0215	0.0036	35.3	2.80E-09	-0.0182	0.0377	0.631
rs3115418	T	C	0.0187	0.0036	27.4	1.61E-07	0.0212	0.0375	0.573
rs3218116	C	T	0.0181	0.0041	19.7	9.02E-06	-0.0188	0.0431	0.664
rs329124	A	G	0.0138	0.0036	14.7	1.23E-04	-0.0170	0.0374	0.652
rs34342129	T	C	0.0147	0.0036	17.2	3.39E-05	-0.0136	0.0372	0.717
rs34399632	G	A	0.0240	0.0041	34.8	3.61E-09	0.0352	0.0435	0.421
rs34553878	G	A	0.0273	0.0057	22.7	1.94E-06	-0.0173	0.0608	0.777
rs35375873	G	C	0.0299	0.0056	28.5	9.44E-08	-0.1177	0.0584	0.045

SNP	Effect allele	Other allele	SNP-smoking association				SNP-LADA association		
			BETA	SE	F statistics	*P*	BETA	SE	*P*
rs35656245	A	G	0.0157	0.0040	15.6	7.97E-05	0.0490	0.0420	0.245
rs357304	C	T	0.0207	0.0040	26.5	2.66E-07	-0.0362	0.0415	0.386
rs359431	C	T	0.0176	0.0036	24.3	8.24E-07	0.0265	0.0375	0.482
rs3740977	C	T	0.0197	0.0048	17.0	3.78E-05	0.0089	0.0500	0.859
rs3764351	G	A	0.0179	0.0038	22.8	1.78E-06	0.0709	0.0390	0.071
rs3800227	G	A	0.0228	0.0041	31.6	1.93E-08	0.0581	0.0431	0.180
rs3811038	C	T	0.0190	0.0040	23.1	1.58E-06	-0.0355	0.0405	0.384
rs3820277	G	T	0.0177	0.0036	24.7	6.64E-07	0.0777	0.0372	0.038
rs3847244	T	C	0.0216	0.0036	36.7	1.34E-09	0.0275	0.0375	0.466
rs3850736	G	C	0.0174	0.0036	24.0	9.45E-07	-0.0333	0.0373	0.375
rs3934797	G	A	0.0243	0.0046	27.6	1.53E-07	0.0188	0.0493	0.704
rs4044321	A	G	0.0278	0.0037	56.3	6.08E-14	0.0584	0.0388	0.135
rs4140932	T	A	0.0158	0.0036	19.4	1.09E-05	0.0037	0.0373	0.921
rs42417	T	C	0.0237	0.0038	38.4	5.80E-10	-0.0024	0.0405	0.954
rs4310804	C	G	0.0212	0.0041	26.9	2.11E-07	0.0582	0.0426	0.175
rs4326350	C	G	0.0164	0.0036	21.1	4.28E-06	-0.0053	0.0469	0.911
rs4476253	G	A	0.0169	0.0042	16.3	5.39E-05	0.0646	0.0440	0.145
rs4543050	T	A	0.0260	0.0046	32.2	1.39E-08	0.0471	0.0481	0.330
rs4674993	A	G	0.0252	0.0044	32.3	1.32E-08	0.0620	0.0477	0.196
rs4727189	C	T	0.0166	0.0038	19.5	1.02E-05	0.0115	0.0393	0.770
rs4752018	A	C	0.0208	0.0042	24.1	9.34E-07	-0.0564	0.0445	0.208
rs4759229	G	A	0.0206	0.0037	30.3	3.62E-08	-0.0963	0.0386	0.013
rs4785187	A	G	0.0202	0.0043	22.5	2.06E-06	-0.1100	0.0453	0.016
rs4790874	T	C	0.0192	0.0036	29.0	7.28E-08	-0.0088	0.0372	0.814
rs4818005	G	A	0.0166	0.0038	19.2	1.15E-05	0.0166	0.0373	0.657
rs4822102	C	T	0.0185	0.0036	25.7	3.97E-07	0.1106	0.0379	0.004
rs4837631	C	T	0.0181	0.0036	25.6	4.10E-07	0.0859	0.0375	0.023
rs4886207	T	C	0.0166	0.0037	20.2	7.01E-06	0.0066	0.0384	0.864
rs4912332	T	C	0.0168	0.0036	22.3	2.34E-06	0.0448	0.0373	0.233
rs540860	G	A	0.0187	0.0036	27.3	1.75E-07	0.0226	0.0373	0.547
rs55913542	T	G	0.0165	0.0047	12.5	4.00E-04	0.0619	0.0498	0.216
rs56208390	G	A	0.0239	0.0055	19.2	1.20E-05	-0.0257	0.0557	0.647
rs56367474	C	T	0.0182	0.0039	22.2	2.46E-06	0.0021	0.0412	0.961
rs56902655	T	G	0.0243	0.0051	22.4	2.19E-06	0.0474	0.0552	0.393
rs58400863	G	A	0.0188	0.0037	25.4	4.75E-07	-0.0236	0.0393	0.550
rs586699	G	A	0.0191	0.0036	28.7	8.61E-08	0.0095	0.0371	0.799
rs59537158	T	C	0.0236	0.0043	30.0	4.28E-08	0.0443	0.0460	0.339
rs6011779	C	T	0.0161	0.0045	12.7	3.63E-04	0.0025	0.0481	0.960
rs6073075	T	A	0.0211	0.0047	20.1	7.37E-06	0.0680	0.0513	0.187
rs60833441	A	G	0.0144	0.0036	16.3	5.47E-05	-0.0400	0.0370	0.282
rs61533748	C	T	0.0190	0.0037	27.2	1.85E-07	0.0259	0.0379	0.497
rs61886926	C	T	0.0181	0.0036	24.6	7.00E-07	0.0017	0.0383	0.964
rs619087	G	A	0.0165	0.0036	21.0	4.52E-06	0.0149	0.0376	0.694
rs61959481	G	A	0.0226	0.0043	27.1	1.91E-07	0.0044	0.0453	0.923
rs62007780	G	T	0.0165	0.0036	20.9	4.83E-06	0.0144	0.0376	0.703

SNP	Effect allele	Other allele	SNP-smoking association				SNP-LADA association		
			BETA	SE	F statistics	*P*	BETA	SE	*P*
rs62052916	A	T	0.0328	0.0066	24.3	8.07E-07	0.0517	0.0743	0.489
rs62180324	G	A	0.0182	0.0043	17.8	2.47E-05	-0.0027	0.0461	0.953
rs62193862	A	G	0.0236	0.0059	16.0	6.32E-05	0.0265	0.0607	0.664
rs62340589	C	G	0.0207	0.0044	22.5	2.09E-06	0.0823	0.0451	0.069
rs6265	C	T	0.0318	0.0046	48.2	3.77E-12	0.0489	0.0470	0.301
rs6437769	T	C	0.0172	0.0036	22.9	1.71E-06	0.0108	0.0380	0.778
rs6438436	T	C	0.0295	0.0045	42.4	7.25E-11	0.0024	0.0478	0.960
rs644740	C	T	0.0154	0.0036	18.7	1.55E-05	0.0151	0.0370	0.686
rs6452785	C	T	0.0266	0.0036	55.8	8.03E-14	0.0475	0.0378	0.211
rs6497840	A	G	0.0179	0.0041	19.0	1.30E-05	0.0774	0.0410	0.061
rs67050670	A	G	0.0203	0.0043	22.7	1.90E-06	0.0297	0.0443	0.505
rs6731872	G	T	0.0352	0.0047	55.4	9.64E-14	-0.0029	0.0491	0.954
rs6750529	T	C	0.0224	0.0041	30.4	3.54E-08	0.0214	0.0437	0.627
rs6756212	C	T	0.0360	0.0036	102.0	6.25E-24	-0.0724	0.0368	0.050
rs67777803	G	T	0.0249	0.0048	27.4	1.65E-07	0.0894	0.0498	0.074
rs6782116	C	T	0.0150	0.0036	17.2	3.37E-05	0.0090	0.0381	0.814
rs6874731	G	T	0.0175	0.0036	24.2	8.63E-07	0.0404	0.0376	0.285
rs6948707	G	T	0.0225	0.0036	38.9	4.50E-10	-0.0538	0.0373	0.151
rs6968380	G	A	0.0195	0.0038	25.9	3.56E-07	0.0020	0.0398	0.961
rs6993429	C	A	0.0193	0.0036	29.1	6.82E-08	0.0224	0.0372	0.549
rs7024924	C	T	0.0258	0.0047	30.2	3.96E-08	0.0604	0.0502	0.232
rs7072776	A	G	0.0260	0.0040	42.9	5.81E-11	-0.0789	0.0417	0.060
rs7134009	T	C	0.0155	0.0041	14.0	1.87E-04	-0.0383	0.0413	0.356
rs71367544	T	C	0.0210	0.0044	23.1	1.55E-06	-0.0498	0.0491	0.313
rs71592686	C	T	0.0235	0.0040	34.9	3.49E-09	0.0110	0.0422	0.796
rs71602617	C	T	0.0152	0.0045	11.2	8.26E-04	0.0537	0.0438	0.222
rs7192140	T	C	0.0157	0.0036	19.6	9.64E-06	0.0458	0.0370	0.219
rs72780746	T	C	0.0293	0.0047	38.7	4.90E-10	0.0208	0.0484	0.670
rs72789626	T	A	0.0321	0.0052	38.2	6.39E-10	-0.0332	0.0533	0.536
rs72898831	A	G	0.0281	0.0050	32.3	1.33E-08	0.1217	0.0515	0.019
rs72938304	G	A	0.0243	0.0056	18.9	1.41E-05	0.0388	0.0570	0.498
rs73008357	A	C	0.0192	0.0057	11.4	7.30E-04	0.0004	0.0541	0.994
rs7333559	G	A	0.0252	0.0043	33.7	6.53E-09	-0.0071	0.0471	0.882
rs748832	G	A	0.0198	0.0037	29.0	7.12E-08	-0.0070	0.0383	0.857
rs7505855	C	T	0.0190	0.0036	27.6	1.45E-07	-0.0093	0.0379	0.806
rs75674569	G	A	0.0234	0.0060	15.0	1.05E-04	-0.0902	0.0643	0.163
rs7585579	G	C	0.0224	0.0037	36.1	1.88E-09	0.0402	0.0372	0.283
rs75919030	T	C	0.0194	0.0040	23.3	1.37E-06	-0.0867	0.0409	0.035
rs7600835	G	A	0.0150	0.0037	16.2	5.64E-05	-0.0023	0.0396	0.954
rs7631379	C	T	0.0219	0.0044	24.7	6.58E-07	-0.0530	0.0479	0.271
rs7657022	G	A	0.0164	0.0036	21.2	4.06E-06	0.0544	0.0370	0.144
rs76841737	C	G	0.0267	0.0058	21.6	3.34E-06	-0.1466	0.0612	0.017
rs7696257	A	G	0.0154	0.0037	17.5	2.89E-05	-0.0122	0.0390	0.757
rs77215829	A	C	0.0262	0.0052	25.3	4.92E-07	0.0234	0.0569	0.683
rs7743165	G	T	0.0193	0.0036	29.4	5.76E-08	-0.0291	0.0369	0.432

SNP	Effect allele	Other allele	SNP-smoking association				SNP-LADA association		
			BETA	SE	F statistics	*P*	BETA	SE	*P*
rs7802996	C	T	0.0203	0.0048	18.1	2.14E-05	0.0323	0.0507	0.526
rs7809303	G	A	0.0215	0.0038	32.3	1.30E-08	0.0135	0.0396	0.735
rs7836565	C	T	0.0160	0.0040	16.4	5.08E-05	-0.0073	0.0413	0.861
rs7867822	A	G	0.0156	0.0038	17.1	3.54E-05	0.0273	0.0387	0.483
rs7920501	T	A	0.0188	0.0036	27.7	1.41E-07	-0.0225	0.0372	0.548
rs7921378	G	C	0.0255	0.0036	51.2	8.26E-13	-0.0642	0.0370	0.085
rs7929518	G	A	0.0242	0.0043	32.0	1.56E-08	0.0166	0.0438	0.706
rs7943721	G	A	0.0224	0.0047	22.9	1.70E-06	0.0468	0.0495	0.348
rs7969559	A	G	0.0244	0.0040	37.9	7.31E-10	0.0988	0.0407	0.016
rs8005334	G	T	0.0178	0.0037	23.0	1.63E-06	0.0179	0.0389	0.646
rs8027457	C	T	0.0188	0.0036	28.0	1.20E-07	0.0633	0.0373	0.092
rs8096225	C	A	0.0137	0.0039	12.5	4.08E-04	-0.0105	0.0407	0.797
rs910912	T	C	0.0219	0.0041	29.3	6.17E-08	-0.0081	0.0426	0.850
rs925524	G	A	0.0141	0.0039	12.9	3.30E-04	-0.0702	0.0408	0.087
rs9288999	A	G	0.0179	0.0040	19.8	8.79E-06	-0.0238	0.0429	0.582
rs9302604	G	A	0.0180	0.0036	25.1	5.34E-07	0.0418	0.0377	0.271
rs9323328	A	G	0.0175	0.0036	24.1	9.07E-07	-0.0173	0.0372	0.643
rs9331343	T	C	0.0161	0.0036	20.1	7.33E-06	0.0466	0.0402	0.248
rs9423279	C	G	0.0205	0.0037	30.6	3.21E-08	-0.0001	0.0394	0.997
rs951740	A	G	0.0310	0.0037	71.0	3.54E-17	-0.0179	0.0390	0.648
rs9540731	C	T	0.0196	0.0036	30.2	3.85E-08	-0.0207	0.0371	0.578
rs9545155	T	C	0.0155	0.0036	19.0	1.29E-05	-0.0582	0.0372	0.120
rs9627272	G	C	0.0191	0.0036	27.8	1.37E-07	0.0064	0.0387	0.869
rs9787523	T	C	0.0151	0.0036	17.5	2.87E-05	-0.0141	0.0378	0.710
rs9826984	G	A	0.0152	0.0036	18.1	2.07E-05	-0.0623	0.0373	0.097
rs9841807	T	C	0.0162	0.0040	16.6	4.60E-05	0.0566	0.0421	0.181
rs9850597	G	A	0.0179	0.0046	15.1	9.96E-05	-0.0142	0.0495	0.776
rs986714	A	T	0.0137	0.0036	14.7	1.25E-04	-0.0068	0.0376	0.858
rs9922607	C	T	0.0241	0.0045	29.2	6.44E-08	0.0248	0.0483	0.609

ESM Table 2. Odds ratios (OR) with 95% confidence intervals (CI) for combinations of snus use and HLA genotypes in the risk of LADA, and attributable proportions due to interaction (AP) with 95% CI*.

**Snus use**	**HLA genotype**	**Cases (n)**	**Controls (n)**	**Model 1 OR (95% CI)**	**Model 2 OR (95% CI)**
Ever snus use	*High-risk*				
-	-	133	542	1	1
+	-	26	60	1.36 (0.76, 2.42)	1.61 (0.87, 3.00)
-	+	190	267	2.91 (2.15, 3.95)	3.28 (2.38, 4.53)
+	+	53	37	4.03 (2.35, 6.90)	5.20 (2.93, 9.21)
				**AP (95% CI)**	0.25 (-0.19, 0.69)
Current snus use	*High-risk*				
-	-	142	568	1	1
+	-	17	34	1.47 (0.72, 3.02)	1.66 (0.78, 3.57)
-	+	205	284	2.85 (2.12, 3.82)	3.20 (2.34, 4.37)
+	+	38	20	5.33 (2.76, 10.29)	6.65 (3.31, 13.36)
				**AP (95% CI)**	0.42 (-0.01, 0.85)
≥15 box-years	*High-risk*				
-	-	147	592	1	1
+	-	12	10	2.65 (0.98, 7.19)	2.54 (0.84, 7.65)
-	+	230	299	3.00 (2.26, 3.99)	3.34 (2.47, 4.51)
+	+	13	5	6.10 (1.90, 19.58)	8.18 (2.40, 27.95)
				**AP (95% CI)**	0.40 (-0.40, 1.19)

Model 1 adjusted for age and sex.
Model 2 adjusted for age, sex, BMI, smoking, educational level, and alcohol consumption.
*Case–control (ESTRID) data only.

ESM Table 3. Odds ratios (OR) with 95% confidence intervals (CI) for combinations of tobacco use and HLA genotypes in the risk of LADA, and attributable proportions due to interaction (AP) with 95% CI (ESTRID).

**Tobacco use**	**HLA genotype**	**Cases (n)**	**Controls (n)**	**Model 1 OR (95% CI)**	**Model 2 OR (95% CI)**
Ever tobacco use	*High-risk*				
-	-	64	274	1	1
+	-	95	328	1.21 (0.81, 1.83)	1.29 (0.84, 1.97)
-	+	101	132	3.18 (2.05, 4.93)	3.35 (2.12, 5.30)
+	+	142	172	3.39 (2.26, 5.07)	4.14 (2.70, 6.35)
				**AP (95% CI)**	0.12 (-0.25, 0.49)
Current tobacco use	*High-risk*				
-	-	109	466	1	1
+	-	50	136	1.45 (0.93, 2.26)	1.54 (0.96, 2.47)
-	+	173	231	3.05 (2.19, 4.25)	3.31 (2.34, 4.68)
+	+	70	73	3.92 (2.50, 6.13)	4.94 (3.07, 7.95)
				**AP (95% CI)**	0.22 (-0.15, 0.59)

Model 1 adjusted for age and sex.
Model 2 adjusted for age, sex, BMI, educational level, and alcohol consumption.

ESM Table 4. Associations of tobacco use with levels of GADA, HOMA-IR and HOMA-B in LADA and type 2 diabetes*.

	GADA			HOMA-IR			HOMA-B		
	**β**	**%**	***p***	**β**	**%**	***p***	**β**	**%**	***p***
					**LADA**				
**Smoking**									
Never	0	–	–	0	–	–	0	–	–
Former	-0.1408	-13.1	0.5025	0.1434	15.4	0.0855	0.1511	16.3	0.0811
Current	0.2983	34.8	0.2175	0.1881	20.7	0.0503	0.1447	15.6	0.1464
<15 pack-years	0.0048	0.5	0.9829	0.0784	8.2	0.3800	0.0644	6.7	0.4873
≥15 pack-years	0.0632	6.5	0.7740	0.2344	26.4	**0.0064**	0.2227	24.9	**0.0125**
Per 5 pack-years	0.0221	2.2	0.5213	0.0276	2.8	**0.0488**	0.0339	3.4	**0.0197**
**Snus use**									
Never	0	–	–	0	–	–	0	–	–
Former	-0.2378	-21.2	0.5464	0.0876	9.2	0.5760	0.0511	5.2	0.7532
Current	-0.2122	-19.1	0.4752	0.0325	3.3	0.7915	0.0665	6.9	0.6019
<15 box-years	-0.0203	-2.0	0.9458	0.0252	2.6	0.8334	0.0555	5.7	0.6547
≥15 box-years	-0.5884	-44.5	0.1209	0.1088	11.5	0.5005	0.0724	7.5	0.6658
Per 5 box-years	-0.0822	-7.9	0.2608	0.0259	2.6	0.3859	0.0148	1.5	0.6336
**Tobacco use**									
Never	0	–	–	0	–	–	0	–	–
Former	-0.0763	-7.3	0.7282	0.1509	16.3	0.0809	0.1117	11.8	0.2139
Current	0.0372	3.8	0.8710	0.1830	20.1	**0.0443**	0.1257	13.4	0.1836
					**Type 2 diabetes**				
**Smoking**									
Never	–	–	–	0		0	0		0
Former	–	–	–	0.0489	5.0	0.1189	0.0272	2.8	0.3814
Current	–	–	–	0.0646	6.7	0.0920	0.0788	8.2	**0.0382**
<15 pack-years	–	–	–	0.0321	3.3	0.3630	0.0350	3.6	0.3863
≥15 pack-years	–	–	–	0.0700	7.3	**0.0301**	0.0570	5.9	0.0877
Per 5 pack-years	–	–	–	0.0087	0.9	**0.0291**	0.0064	0.6	0.1055
**Snus use**	–								
Never	–	–	–	0		0	0		0
Former	–	–	–	0.0951	10.0	0.0755	-0.1115	-10.6	**0.0354**
Current	–	–	–	0.0456	4.7	0.3150	-0.0038	-0.4	0.9329
<15 box-years	–	–	–	0.0210	2.1	0.6205	-0.0139	-1.4	0.7413
≥15 box-years	–	–	–	0.1754	19.2	**0.0041**	-0.1285	-12.1	**0.0342**
Per 5 box-years	–	–	–	0.0275	2.8	**0.0099**	-0.0262	-2.6	**0.0129**
**Tobacco use**	–								
Never	–	–	–	0		0	0		0
Former	–	–	–	0.0846	8.8	0.0101	0.0194	12.0	0.5521
Current	–	–	–	0.0985	10.4	**0.0062**	0.0386	3.9	0.2794

Models adjusted for age, sex, BMI, educational level, and alcohol consumption. Snus analyses additionally adjusted for smoking.
*Case−control (ESTRID) data only.
Information on GADA available for LADA (n=583). HOMA-IR and HOMA-B available for LADA (n=475) and type 2 diabetes (n=1752).

ESM Table 5. Odds ratios (OR) with 95% confidence intervals (CI) for the association between smoking and LADA and type 2 diabetes (ESTRID).

	LADA				Type 2 diabetes			
	Cases (n)	Controls (n)	Model 1 OR (95% CI)	Model 2 OR (95% CI)	Cases (n)	Controls (n)	Model 1 OR (95% CI)	Model 2 OR (95% CI)
**Smoking**								
Never	271	1467	1	1	782	1467	1	1
Former	186	964	1.08 (0.86, 1.34)	1.10 (0.87, 1.39)	850	964	1.36 (1.18, 1.57)	1.48 (1.24, 1.77)
Current	136	605	1.40 (1.10, 1.79)	1.52 (1.18, 1.96)	406	605	1.39 (1.17, 1.65)	1.70 (1.37, 2.09)
Ever	322	1569	1.20 (0.99, 1.45)	1.25 (1.03, 1.53)	1256	1569	1.37 (1.20, 1.56)	1.56 (1.33, 1.82)
**Intensity (current)**								
Non	457	2431	1	1	1632	2431	1	1
<20 cigarettes per day	97	497	1.21 (0.93, 1.56)	1.33 (1.02, 1.75)	304	497	1.18 (0.99, 1.41)	1.51 (1.22, 1.87)
≥20 cigarettes per day	33	98	1.91 (1.23, 2.96)	1.77 (1.11, 2.80)	89	98	1.31 (0.94, 1.81)	1.17 (0.79, 1.73)
Per 5 cigarettes	593	3036	1.13 (1.05, 1.22)	1.14 (1.05, 1.23)	2038	3036	1.10 (1.04, 1.16)	1.11 (1.04, 1.19)
**Pack-years (ever)**								
Never	271	1467	1	1	782	1467	1	1
<15 pack-years	155	932	1.01 (0.80, 1.26)	1.10 (0.87, 1.40)	507	932	1.04 (0.88, 1.21)	1.26 (1.04, 1.52)
≥15 pack-years	167	637	1.47 (1.17, 1.86)	1.47 (1.15, 1.88)	749	637	1.82 (1.56, 2.12)	1.93 (1.60, 2.34)
Per 5 pack-years	593	3036	1.05 (1.01, 1.09)	1.04 (1.00, 1.08)	2038	3036	1.10 (1.07, 1.12)	1.09 (1.06, 1.12)

Model 1 adjusted for age and sex.
Model 2 adjusted for age, sex, BMI, educational level, and alcohol consumption.

ESM Table 6. Hazard ratios (HR) with 95% confidence intervals (CI) for the association between smoking and LADA and type 2 diabetes (HUNT).

	LADA				Type 2 diabetes			
	**Cases (n)**	**Person-years**	**Model 1 HR (95% CI)**	**Model 2 HR (95% CI)**	**Cases (n)**	**Person-years**	**Model 1 HR (95% CI)**	**Model 2 HR (95% CI)**
**Smoking**								
Never	107	729,291	1	1	1499	729,291	1	1
Former	83	465,674	1.04 (0.78, 1.40)	1.04 (0.77, 1.40)	1234	465,674	1.06 (0.98, 1.14)	1.01 (0.94, 1.09)
Current	55	501,537	0.79 (0.57, 1.09)	0.99 (0.71, 1.38)	993	501,537	1.06 (0.98, 1.15)	1.34 (1.24, 1.46)
Ever	138	967,211	0.92 (0.71, 1.19)	1.02 (0.78, 1.32)	2227	967,211	1.06 (0.99, 1.13)	1.14 (1.06, 1.22)
**Intensity (current)**								
Non	190	1 194,965	1	1	2733	1 194,965	1	1
<20 cigarettes per day	30	358,396	0.64 (0.43, 0.96)	0.84 (0.55, 1.27)	616	358,396	0.99 (0.90, 1.09)	1.32 (1.20, 1.46)
≥20 cigarettes per day	22	112,955	1.21 (0.76, 1.91)	1.34 (0.84, 2.14)	336	112,955	1.35 (1.20, 1.52)	1.50 (1.33, 1.69)
Per 5 cigarettes	245	1 696,503	0.89 (0.78, 1.01)	0.95 (0.84, 1.07)	3726	1 696,503	1.01 (0.98, 1.04)	1.07 (1.04, 1.10)
**Pack-years (ever)**								
Never	107	729,291	1	1	1499	729,291	1	1
<15 pack-years	74	527,176	0.99 (0.74, 1.34)	1.12 (0.83, 1.51)	956	527,176	0.98 (0.90, 1.06)	1.09 (1.01, 1.19)
≥15 pack-years	43	276,814	0.86 (0.60, 1.23)	0.94 (0.65, 1.35)	911	276,814	1.28 (1.17, 1.39)	1.37 (1.25, 1.49)
Per 5 pack-years	245	1 696,503	0.99 (0.93, 1.06)	1.00 (0.94, 1.07)	3726	1 696,503	1.05 (1.03, 1.06)	1.05 (1.04, 1.07)

Model 1 adjusted for age and sex.
Model 2 adjusted for age, sex, BMI, educational level, and alcohol consumption.

ESM Table 7. Hazard ratios (HR) with 95% confidence intervals (CI) for the association between smoking and the risk of LADA and type 2 diabetes by HUNT wave.

Smoking	LADA				Type 2 diabetes			
	Cases	Person-years	Model 1 HR (95% CI)	Model 2 HR (95% CI)	Cases	Person-years	Model 1 HR (95% CI)	Model 2 HR (95% CI)
HUNT1								
Never	56	271,021	1	1	502	271,021	1	1
Former	37	154,586	1.05 (0.67, 1.64)	1.07 (0.68, 1.69)	322	154,586	1.03 (0.88, 1.19)	1.06 (0.91, 1.24)
Current	9	222,222	0.23 (0.11, 0.46)	0.27 (0.13, 0.58)	317	222,222	0.96 (0.82, 1.11)	1.26 (1.08, 1.47)
Ever	46	376,809	0.60 (0.40, 0.91)	0.70 (0.46, 1.08)	639	376,809	0.99 (0.87, 1.12)	1.15 (1.01, 1.31)
HUNT2								
Never	28	284,644	1	1	562	284,644	1	1
Former	25	179,822	1.20 (0.70, 2.07)	1.18 (0.68, 2.04)	479	179,822	1.09 (0.97, 1.24)	1.04 (0.92, 1.18)
Current	25	187,280	1.32 (0.77, 2.28)	1.63 (0.94, 2.84)	406	187,280	1.12 (0.98, 1.27)	1.40 (1.23, 1.60)
Ever	50	367,102	1.26 (0.79, 2.01)	1.37 (0.85, 2.19)	885	367,102	1.11 (0.99, 1.23)	1.18 (1.06, 1.31)
HUNT3								
Never	23	169,227	1	1	421	169,227	1	1
Former	20	126,589	1.05 (0.57, 1.92)	0.97 (0.53, 1.80)	421	126,589	1.10 (0.96, 1.26)	1.06 (0.92, 1.22)
Current	21	87,097	1.78 (0.98, 3.24)	1.84 (1.01, 3.36)	262	87,097	1.27 (1.08, 1.48)	1.42 (1.22, 1.67)
Ever	41	213,686	1.33 (0.80, 2.24)	1.29 (0.77, 2.18)	683	213,686	1.16 (1.03, 1.31)	1.18 (1.04, 1.33)

Model 1 adjusted for age and sex.
Model 2 adjusted for age, sex, BMI, educational level, and alcohol consumption.

ESM Table 8. Odds ratios (OR) with 95% confidence intervals (CI) for combinations of smoking and HLA genotypes in the risk of LADA, and attributable proportions due to interaction (AP) with 95% CI (ESTRID).

**Smoking**	**HLA genotype**	**Cases (n)**	**Controls (n)**	**Model 1 OR (95% CI)**	**Model 2 OR (95% CI)**
Ever smoking	*High-risk*				
-	-	71	285	1	1
+	-	88	317	1.16 (0.78−1.75)	1.17 (0.77−1.79)
-	+	116	137	3.25 (2.14−4.95)	3.38 (2.18−5.24)
+	+	127	167	3.16 (2.12−4.71)	3.75 (2.47−5.72)
				**AP (95% CI)**	0.05 (-0.35−0.45)
Current smoking	*High-risk*				
-	-	121	488	1	1
+	-	38	114	1.39 (0.86−2.24)	1.43 (0.86−2.36)
-	+	192	247	2.99 (2.18−4.10)	3.22 (2.31−4.50)
+	+	51	57	3.97 (2.43−6.48)	5.16 (3.07−8.67)
				**AP (95% CI)**	0.29 (-0.08−0.67)
≥15 pack-years[a]	*High-risk*				
-	-	113	474	1	1
+	-	46	128	1.76 (1.12−2.76)	1.64 (1.01−2.64)
-	+	175	234	3.19 (2.29−4.44)	3.38 (2.40−4.77)
+	+	68	70	4.12 (2.62−6.47)	4.82 (2.98−7.77)
				**AP (95% CI)**	0.17 (-0.24−0.57)

Model 1 adjusted for age and sex.
Model 2 adjusted for age, sex, BMI, educational level, and alcohol consumption.
[a]The reference category (<15 pack-years) includes never-smokers.

ESM Table 9. Hazard ratios (HR) with 95% confidence intervals (CI) for combinations of smoking and HLA genotypes in the risk of LADA, and attributable proportions due to interaction (AP) with 95% CI (HUNT).

**Smoking**	**HLA genotype**	**Cases (n)**	**Person-years**	**Model 1 HR (95% CI)**	**Model 2 HR (95% CI)**
Ever smoking	*High-risk*				
-	-	49	425,079	1	1
+	-	63	574,688	0.90 (0.62, 1.32)	1.00 (0.69, 1.47)
-	+	40	177,868	1.96 (1.29, 2.98)	1.92 (1.26, 2.92)
+	+	67	228,292	2.43 (1.68, 3.52)	2.66 (1.83, 3.87)
				**AP (95% CI)**	0.28 (-0.04, 0.59)
Current smoking	*High-risk*				
-	-	87	705,267	1	1
+	-	25	294,500	0.77 (0.49, 1.20)	0.96 (0.61, 1.50)
-	+	78	291,593	2.19 (1.61, 2.97)	2.15 (1.58, 2.92)
+	+	29	114,567	2.28 (1.50, 3.48)	2.82 (1.84, 4.31)
				**AP (95% CI)**	0.25 (-0.10, 0.61)
≥15 pack-years[a]	*High-risk*				
-	-	91	815,808	1	1
+	-	21	183,959	0.79 (0.47, 1.32)	0.82 (0.49, 1.38)
-	+	84	334,265	2.35 (1.72, 3.20)	2.30 (1.69, 3.13)
+	+	23	71,896	2.59 (1.62, 4.14)	2.67 (1.67, 4.28)
				**AP (95% CI)**	0.21 (-0.20, 0.62)

Model 1 adjusted for age and sex.
Model 2 adjusted for age, sex, BMI, educational level, and alcohol consumption.
[a]The reference category (<15 pack-years) includes never-smokers.

ESM Table 10. Odds ratios (OR) with 95% confidence intervals (CI) for the association between smoking and LADA and type 2 diabetes, using ESTRID internal controls.

	**LADA**					**Type 2 diabetes**				
	**Cases (n)**	**Controls (n)**	**Model 1 OR (95% CI)**	**Model 2 OR (95% CI)**	**Model 3 OR (95% CI)**	**Cases (n)**	**Controls (n)**	**Model 1 OR (95% CI)**	**Model 2 OR (95% CI)**	**Model 3 OR (95% CI)**
**Smoking**										
Never	271	1198	1	1	1	782	1198	1	1	
Former	186	718	1.08 (0.87, 1.34)	1.01 (0.81, 1.26)	1.00 (0.80, 1.25)	850	718	1.42 (1.23, 1.64)	1.37 (1.16, 1.62)	1.41 (1.19, 1.67)
Current	136	445	1.29 (1.02, 1.64)	1.34 (1.05, 1.71)	1.29 (1.01, 1.66)	406	445	1.33 (1.12, 1.58)	1.50 (1.23, 1.83)	1.48 (1.20, 1.82)
Ever	322	1163	1.17 (0.97, 1.40)	1.13 (0.94, 1.37)	1.11 (0.92, 1.35)	1256	1163	1.39 (1.22, 1.58)	1.42 (1.22, 1.64)	1.43 (1.23, 1.67)
**Intensity (current)**										
Non	457	1916	1	1	1	1632	1916	1	1	1
<20 cigarettes per day	97	344	1.17 (0.91, 1.51)	1.27 (0.98, 1.64)	1.23 (0.95, 1.60)	304	344	1.10 (0.92, 1.32)	1.32 (1.07, 1.63)	1.27 (1.02, 1.57)
≥20 cigarettes per day	33	71	1.84 (1.19, 2.83)	1.82 (1.16, 2.84)	1.75 (1.10, 2.77)	89	71	1.49 (1.07, 2.09)	1.52 (1.03, 2.24)	1.56 (1.04, 2.34)
**Pack-years (ever)**										
Never	271	1198	1	1	1	782	1198	1	1	1
<15 pack-years	155	641	1.04 (0.83, 1.30)	1.06 (0.84, 1.32)	1.03 (0.82, 1.30)	507	641	1.11 (0.95, 1.30)	1.21 (1.01, 1.45)	1.22 (1.01, 1.46)
≥15 pack-years	167	522	1.32 (1.06, 1.66)	1.22 (0.97, 1.54)	1.21 (0.95, 1.53)	749	522	1.71 (1.47, 1.99)	1.64 (1.37, 1.95)	1.67 (1.39, 2.00)
Per 5 pack-years	593	2361	1.03 (1.00, 1.06)	1.02 (0.98, 1.05)	1.02 (0.98, 1.05)	2038	2361	1.08 (1.06, 1.11)	1.07 (1.04, 1.10)	1.07 (1.05, 1.10)

Model 1 adjusted for age and sex.
Model 2 adjusted for age, sex, BMI, educational level, and alcohol consumption.
Model 3 adjusted as Model 2 and additionally for family history of diabetes.

ESM Table 11. Odds ratios (OR) with 95% confidence intervals (CI) for the association between snus use and LADA and type 2 diabetes, restricted to only men.

	LADA				Type 2 diabetes			
	Cases (n)	Controls (n)	Model 1 OR (95% CI)	Model 2 OR (95% CI)	Cases (n)	Controls (n)	Model 1 OR (95% CI)	Model 2 OR (95% CI)
**Snus use**								
Never	218	607	1	1	842	607	1	1
Former	32	91	1.06 (0.68, 1.64)	1.14 (0.72, 1.82)	152	91	1.33 (1.00, 1.78)	1.22 (0.86, 1.74)
Current		145	1.22 (0.87, 1.73)	1.27 (0.87, 1.85)	228	145	1.53 (1.20, 1.96)	1.65 (1.23, 2.23)
Ever	96	236	1.16 (0.87, 1.56)	1.22 (0.89, 1.68)	380	236	1.45 (1.18, 1.77)	1.47 (1.14, 1.88)
**Intensity (current)**								
Non	250	698	1	1	994	698	1	1
<7 boxes per week	53	131	1.11 (0.77, 1.59)	1.19 (0.81, 1.76)	190	131	1.33 (1.03, 1.72)	1.55 (1.14, 2.12)
≥7 boxes per week	9	12	2.31 (0.95, 5.62)	1.63 (0.59, 4.55)	32	12	2.88 (1.45, 5.71)	2.11 (0.95, 4.65)
**Box-years (ever)**								
Never	218	607	1	1	842	607	1	1
<15 box-years	62	181	0.95 (0.68, 1.33)	1.05 (0.73, 1.50)	263	181	1.25 (1.00, 1.57)	1.33 (1.01, 1.75)
≥15 box-years	34	55	1.91 (1.20, 3.05)	1.81 (1.08, 3.00)	117	55	2.15 (1.52, 3.05)	1.92 (1.26, 2.92)
Per 5 box-years	314	843	1.11 (1.02, 1.22)	1.10 (0.99, 1.21)	1222	843	1.16 (1.09, 1.24)	1.12 (1.03, 1.21)

Model 1 adjusted for age and sex.
Model 2 adjusted for age, sex, BMI, smoking, educational level, and alcohol consumption.

ESM Table 12. Odds ratios (OR) with 95% confidence intervals (CI) for the association between snus use and LADA and type 2 diabetes, restricted to never-smokers.

	**LADA**				**Type diabetes**			
	**Cases (n)**	**Controls (n)**	**Model 1 OR (95% CI)**	**Model 2 OR (95% CI)**	**Cases (n)**	**Controls (n)**	**Model 1 OR (95% CI)**	**Model 2 OR (95% CI)**
**Snus use**								
Never	246	1379	1	1	700	1379	1	1
Former	8	24	1.43 (0.61, 3.36)	1.59 (0.65, 3.88)	29	24	1.48 (0.81, 2.68)	1.24 (0.58, 2.64)
Current	17	64	0.85 (0.46, 1.58)	0.92 (0.48, 1.77)	53	64	1.33 (0.87, 2.03)	1.73 (1.02, 2.93)
Ever	25	88	0.99 (0.59-1.68)	1.09 (0.62, 1.89)	82	88	1.37 (0.96, 1.97)	1.56 (0.99, 2.46)
**Intensity (current)**								
Non	254	1403	1	1	729	1403	1	1
<7 boxes per week	14	57	0.73 (0.37, 1.42)	0.84 (0.42, 1.68)	41	57	1.05 (0.67, 1.66)	1.53 (0.87, 2.70)
≥7 boxes per week	2	7	0.96 (0.18, 5.02)	0.64 (0.10, 4.23)	11	7	3.51 (1.19, 10.34)	2.11 (0.59, 7.58)
**Box-years (ever)**								
Never	246	1379	1	1	700	1379	1	1
<15 box-years	14	64	0.78 (0.41, 1.50)	0.89 (0.45, 1.74)	36	64	0.91 (0.57, 1.44)	1.19 (0.68, 2.09)
≥15 box-years	11	24	1.52 (0.69, 3.32)	1.58 (0.69, 3.66)	46	24	2.50 (1.44, 4.33)	2.36 (1.19, 4.70)
Per 5 box-years	271	1467	1.05 (0.89, 1.23)	1.04 (0.87, 1.24)	782	1467	1.19 (1.07, 1.31)	1.16 (1.01, 1.32)

Model 1 adjusted for age and sex.
Model 2 adjusted for age, sex, BMI, educational level, and alcohol consumption.

ESM Table 13. Conservative analyses of associations between smoking and LADA/type 2 diabetes in the MR study

Conservative analyses	No. of included SNPs	No. of excluded SNPs	Total no. of SNPs	OR (95% CI)	*p* for risk estimate	*p* for heterogeneity
**LADA**						
1	155	95	250	1.51 (1.08, 2.12)	0.017	0.189
2	152	98	250	1.58 (1.12, 2.23)	0.009	0.206
3	137	113	250	1.62 (1.13, 2.34)	0.009	0.413
**Type 2 diabetes**						
1	154	95	249	1.15 (1.02, 1.28)	0.017	<0.001
2	151	98	249	1.11 (0.99, 1.25)	0.062	<0.001
3	136	113	249	1.10 (0.98, 1.24)	0.111	<0.001

LADA: latent autoimmune diabetes in adults; MR: mendelian randomisation; SNP: single nucleotide polymorphism; OR: odds ratio; CI: confidence interval.

Conservative analysis 1 excluded SNPs associated with any trait (except smoking) at $p<5\times10^{-8}$;

Conservative analysis 2 excluded SNPs excluded by conservative analysis 1 and SNPs associated with diabetes-related traits at $p<5\times10^{-4}$;

Conservative analysis 3 excluded SNPs excluded by conservative analysis 2 and SNPs associated with alcohol-related traits at $p<5\times10^{-4}$

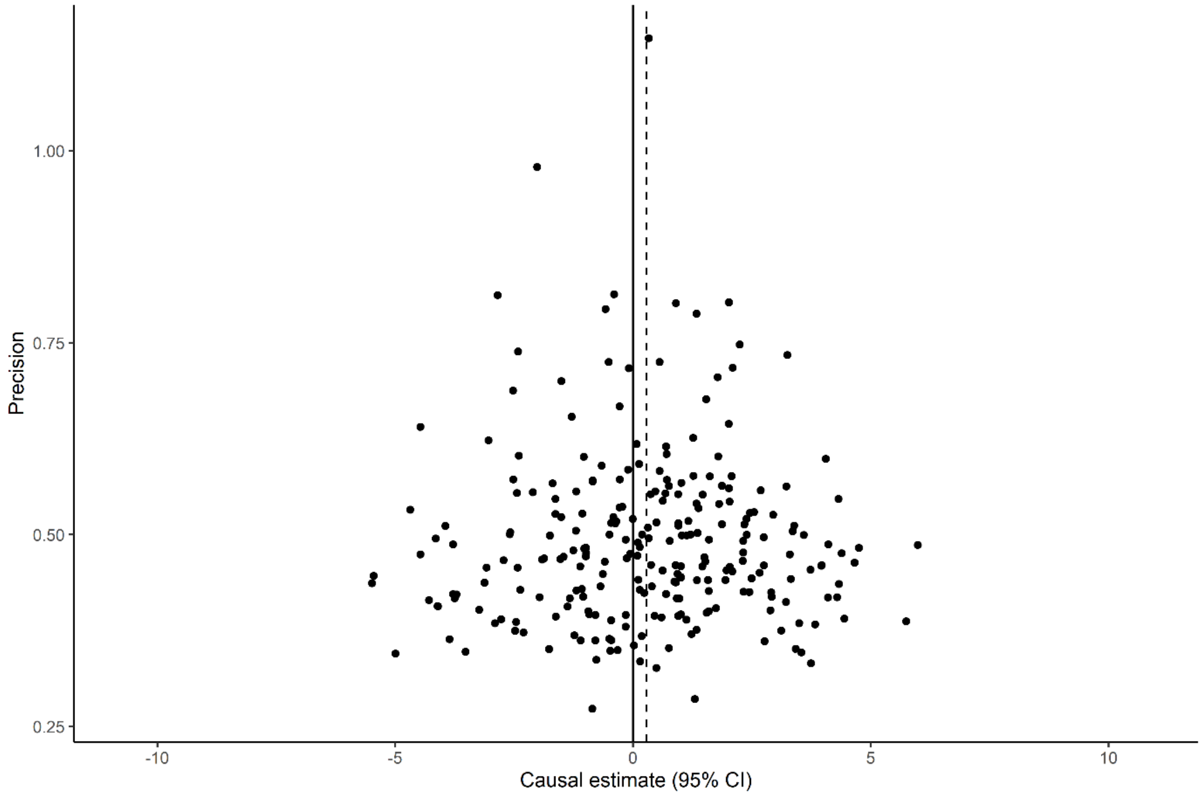

ESM Fig. 1. Funnel plot for the association between smoking and LADA in MR analysis

LADA: latent autoimmune diabetes in adults; MR: Mendelian randomisation

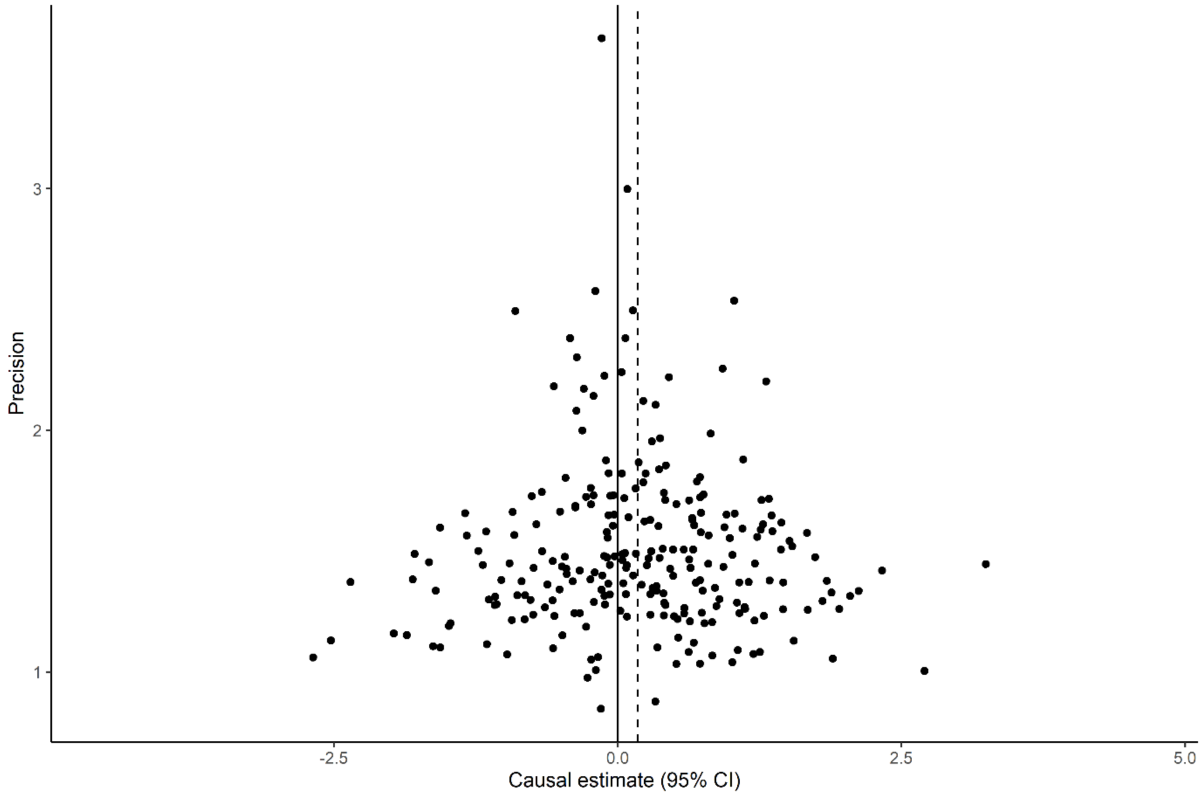

ESM Fig. 2. Funnel plot for the association between smoking and type 2 diabetes in MR analysis

MR: Mendelian randomisation

1. Smith GD, Ebrahim S (2003) “Mendelian randomization”: Can genetic epidemiology contribute to understanding environmental determinants of disease? Int J Epidemiol 32(1):1–22. https://doi.org/10.1093/ije/dyg070
2. Burgess S, Thompson S (2015) Mendelian Randomization: Methods for Using Genetic Variants in Causal Estimation. Chapman and Hall/CRC Press, London, UK
3. Burgess S, Scott RA, Timpson NJ, Smith GD, Thompson SG (2015) Using published data in Mendelian randomization: A blueprint for efficient identification of causal risk factors. Eur J Epidemiol 30(7):543–552. https://doi.org/10.1007/s10654-015-0011-z
4. Burgess S, Bowden J, Dudbridge F, Thompson S (2016) Robust instrumental variable methods using multiple candidate instruments with application to Mendelian randomization., In. Vol 16
5. Liu M, Jiang Y, Wedow R, et al (2019) Association studies of up to 1.2 million individuals yield new insights into the genetic etiology of tobacco and alcohol use. Nat Genet 51(2):237–244. https://doi.org/10.1038/s41588-018-0307-5
6. Zuo L, Tan Y, Li CSR, et al (2016) Associations of rare nicotinic cholinergic receptor gene variants to nicotine and alcohol dependence. Am J Med Genet Part B Neuropsychiatr Genet 171(8):1057–1071. https://doi.org/10.1002/ajmg.b.32476
7. Cousminer DL, Ahlqvist E, Mishra R, et al (2018) First genome-wide association study of latent autoimmune diabetes in adults reveals novel insights linking immune and metabolic diabetes. Diabetes Care 41(11):2396–2403. https://doi.org/10.2337/dc18-1032
8. Scott RA, Scott LJ, Mägi R, et al (2017) An Expanded Genome-Wide Association Study of Type 2 Diabetes in Europeans. Diabetes 66(11):2888–2902. https://doi.org/10.2337/db16-1253
9. Koller M, Stahel W (2011) Sharpening Wald-type inference in robust regression for small samples. Comput Stat Data Anal 55(8):2504–2515
10. Bowden J, Davey Smith G, Haycock PC, Burgess S (2016) Consistent Estimation in Mendelian Randomization with Some Invalid Instruments Using a Weighted Median Estimator. Genet Epidemiol 40(4):304–314. https://doi.org/10.1002/gepi.21965
11. Bowden J, Smith GD, Burgess S (2015) Mendelian randomization with invalid instruments: Effect estimation and bias detection through Egger regression. Int J Epidemiol 44(2):512–525. https://doi.org/10.1093/ije/dyv080
12. Verbanck M, Chen C, Neale B, et al (2018) Detection of widespread horizontal pleiotropy in causal relationships inferred from Mendelian randomization between complex traits and diseases. Nat Genet 50(5):693–698. https://doi.org/10.1038/s41588-018-0099-7.Detection
13. Staley JR, Blackshaw J, Kamat MA, et al (2016) PhenoScanner: A database of human genotype-phenotype associations. Bioinformatics 32(20):3207–3209. https://doi.org/10.1093/bioinformatics/btw373
14. Kamat MA, Blackshaw JA, Young R, et al (2019) PhenoScanner V2: An expanded tool for searching human genotype-phenotype associations. Bioinformatics 35(22):4851–4853. https://doi.org/10.1093/bioinformatics/btz469

15. Bowden J, Fabiola Del Greco M, Minelli C, Smith GD, Sheehan NA, Thompson JR (2016) Assessing the suitability of summary data for two-sample mendelian randomization analyses using MR-Egger regression: The role of the I 2 statistic. Int J Epidemiol 45(6):1961–1974. https://doi.org/10.1093/ije/dyw220

16. Burgess S, Bowden J, Fall T, Ingelsson E, Thompson SG (2017) Sensitivity analyses for robust causal inference from mendelian randomization analyses with multiple genetic variants. Epidemiology 28(1):30–42. https://doi.org/10.1097/EDE.0000000000000559
